# Depression Classification Using Frequent Subgraph Mining Based on Pattern Growth of Frequent Edge in Functional Magnetic Resonance Imaging Uncertain Network

**DOI:** 10.3389/fnins.2022.889105

**Published:** 2022-04-29

**Authors:** Yao Li, Zihao Zhou, Qifan Li, Tao Li, Ibegbu Nnamdi Julian, Hao Guo, Junjie Chen

**Affiliations:** ^1^College of Information and Computer, Taiyuan University of Technology, Taiyuan, China; ^2^College of Mathematics, Taiyuan University of Technology, Taiyuan, China

**Keywords:** frequent subgraph mining, discriminative feature selection, machine learning, classification, fMRI, depression, uncertain brain network

## Abstract

The brain network structure is highly uncertain due to the noise in imaging signals and evaluation methods. Recent works have shown that uncertain brain networks could capture uncertain information with regards to functional connections. Most of the existing research studies covering uncertain brain networks used graph mining methods for analysis; for example, the mining uncertain subgraph patterns (MUSE) method was used to mine frequent subgraphs and the discriminative feature selection for uncertain graph classification (DUG) method was used to select discriminant subgraphs. However, these methods led to a lack of effective discriminative information; this reduced the classification accuracy for brain diseases. Therefore, considering these problems, we propose an approximate frequent subgraph mining algorithm based on pattern growth of frequent edge (unFEPG) for uncertain brain networks and a novel discriminative feature selection method based on statistical index (dfsSI) to perform graph mining and selection. Results showed that compared with the conventional methods, the unFEPG and dfsSI methods achieved a higher classification accuracy. Furthermore, to demonstrate the efficacy of the proposed method, we used consistent discriminative subgraph patterns based on thresholding and weighting approaches to compare the classification performance of uncertain networks and certain networks in a bidirectional manner. Results showed that classification performance of the uncertain network was superior to that of the certain network within a defined sparsity range. This indicated that if a better classification performance is to be achieved, it is necessary to select a certain brain network with a higher threshold or an uncertain brain network model. Moreover, if the uncertain brain network model was selected, it is necessary to make full use of the uncertain information of its functional connection.

## Introduction

Over recent years, the use of neuroimaging technology to investigate the interaction of brain regions has gained has attracted much attention and recognition (Richardson, 2010). The Blood Oxygen Level-Dependent (BOLD) signal is now routinely used as a neurophysiological indicator for resting-state functional magnetic resonance imaging (rs-fMRI) to detect endogenous or spontaneous activity in the brain neurons. According to BOLD signals, a functional connectivity network can be built and then applied to research the pathological mechanisms underlying brain diseases. This theory has been widely applied to the diagnosis of brain diseases, including schizophrenia (Steardo et al., 2020), depression (Sen et al., 2019), attention deficit syndrome (Riaz et al., 2020), and Alzheimer’s disease (Shao et al., 2020).

Recent researchers have stated that uncertainty is inherent in graph data connections and that this is due to problems associated with data acquisition, the accuracy of equipment, and evaluation methods (Yuan et al., 2016; Khan et al., 2018b; de Ridder et al., 2019). These challenges suggest that it is only possible to provide the probability of a link in the graph, rather than precise values. For instance, the acquisition of fMRI data is influenced by a variety of distinct factors, like subject age (Wig, 2017), head movement (Vakamudi et al., 2019), scanning time (Hagler et al., 2019), vasoconstriction (An et al., 2015), heartbeat and respiration (Pinto et al., 2017; Tong et al., 2019), arterial blood pressure (Steiner et al., 2020), and arterial carbon dioxide concentration (Driver et al., 2016; Prokopiou et al., 2018). Moreover, increasing evidence suggests that even in the resting state, the neural activity in the brain still exhibits transient and subtle dynamics (Kudela et al., 2017; Zhao et al., 2020). However, most studies considered that the interaction of brain regions remains unchanged during the resting state, so as to construct a brain functional network. Therefore, they can be concluded that the functional connections between brain regions are highly uncertain if the rs-fMRI data is employed to build the brain network. These functional connections are obtained by considering processing steps, such as the analysis of temporal correlations in spontaneous BOLD signal oscillations, where each edge refers to a probability to calculate the likelihood that the functional connection exists in the brain.

Previous studies have applied traditional brain network analysis based on certain network for the diagnosis of brain diseases (Sporns, 2011, 2018; Farahani et al., 2020; Zhao et al., 2021). This theory claims deciding whether there is an edge between two brain regions; this is resolved using a threshold or a threshold range (Zhou Z. et al., 2020). The employment of binary networks helps to measuring the network properties and diminishing the burden caused by the generation of graphs. However, the employment of the threshold approach to construct a certain network unavoidably results in the loss of uncertain information (Kong et al., 2013; Hamdi et al., 2018; Zhang et al., 2018). Simultaneously, in exiting researches, there is no gold standard for deciding how to choose the optimal threshold for constructing the effective certain network (Garrison et al., 2015).

To settle the issues of threshold selection in traditional network, researchers selected a small range of thresholds to evade sensitivity related to the selection of a threshold (Jie et al., 2014); however, this method may result in incomplete results or even misdirecting results if the network properties are unsteady within a larger threshold range (Graham et al., 2009; Zhang et al., 2018). Based on this problem, some researchers have proposed the minimum spanning tree (MST) method to build brain networks (Jackson and Read, 2010a; Stam et al., 2014). However, the MST may miss the emphasize of low weight connections and clusters in the interaction of the brain regions (Tewarie et al., 2015), in particularly, from loops formed by low weight links (Li et al., 2011). Moreover, MST analysis may be less sensitive to small differences in the signal-to-noise ratio between subjects because the MST was only lied in the rank of the link weights of the strongest network connections (Van Dellen et al., 2018). In addition, although MST analysis is not dependent on the section of the threshold, it is influenced by the network scale. which further effect the classification performance (Van Dellen et al., 2018). In addition, there are other studies that used direct functional connectivity strength as a feature for classification (Zhang et al., 2021). Although this method also effectively avoids the problems caused by threshold selection, it does not construct a brain network and lacks information relating to network topology properties; thus, whether the network is connected or disconnected becomes irrelevant.

Considering above problems, the concept of the uncertain network was introduced to characterize the uncertainty of functional connections (Kong et al., 2013; Cao et al., 2015a,b; Saha et al., 2021). Uncertain networks are based on uncertain graph theory, where each node represents one object and each edge is related to probabilities so that we can quantify the chances that a pair of nodes exit (Khan et al., 2018b; Ke et al., 2020). In neuroimaging, each node in an uncertain network refers to a brain region, and each edge refers to a probabilistic connection; this indicates the likelihood that a functional connection exists in the brain. Over the past few years, uncertain networks have been successfully applied to the field of neuroimaging. For example, Kong et al. (2013) proposed the discriminative feature selection for uncertain graph classification (DUG) algorithm to mine discriminative subgraphs in uncertain brain network using fMRI data and used this to classify Alzheimer’s disease and normal controls. In another study, Cao et al. (2015b) proposed an uncertain graph mining framework based on current data mining techniques and then verified the framework using a bipolar dataset and identified abnormal subgraph patterns in fMRI data. In addition, Saha et al. (2021) reported how to compute a novel concept of betweenness centrality in an uncertain brain network and used subjects with autism to validate the efficacy of the proposed solution.

As an important topological feature of an uncertain network, a “frequent subgraph” represents the connected patterns that appear most often in the network; this is an essential approach for characterize uncertain graph (Zou et al., 2009; Kong and Yu, 2014; Yuan et al., 2016; Chen et al., 2019). This approach not only models the network connectivity patterns around nodes but also capture changes on local areas. That is, subgraph patterns could balance local topological information with global graph topological information (Kong and Yu, 2014; Cao et al., 2015b). Therefore, in the analysis of uncertain brain networks, most researchers usually used subgraph patterns to quantify uncertain brain networks and applied them to explore brain diseases (Kong et al., 2013; Cao et al., 2015b). Specifically, the mining uncertain subgraph patterns (MUSE) algorithm was mainly used to mine the frequent subgraphs of uncertain brain networks, and the DUG method was used to select discriminative subgraphs. Although the MUSE algorithm has been successfully applied to extract frequent subgraphs, a limitation of this algorithm is that the time complexity is quite high (Papapetrou et al., 2011). Therefore, in the present study, we improved on this algorithm and then proposed an approximate algorithm; that is, we developed a frequent subgraph pattern mining algorithm based on pattern growth of frequent edge in an uncertain network (unFEPG). In this algorithm, pattern growth of frequent edge was employed to substitute the original pruning process exploited to frequent subgraphs. This decreased the time consumption of the method and gives an effective solution to the excessive computational cost of the MUSE algorithm which arose from too many subgraph features being extracted.

Previous researchers proposed the DUG method to identify discriminative subgraph features in uncertain graphs based on a statistical index (Kong et al., 2013; Cao et al., 2015b). Specifically, based on the discrimination score function, dynamic programming was used to calculate the probability distribution of each subgraph. Then, combined with the theory of the discrimination score function in a certain graph, the discrimination score (statistical index) of each subgraph was calculated. Based on discrimination score, discriminative subgraphs were selected. The DUG method was able to obtain the discrimination score in an effective manner but also caused excessive computational consumption due to the use of the dynamic programming method. In addition, previous studies reported that the classification accuracy of brain diseases obtained by the DUG method was too low; that is, this method could not effectively extract biomarkers for specific brain diseases (Kong et al., 2013). Thus, in this paper, we propose a novel discriminative feature selection method that is based on the statistical index (dfsSI). Unlike the DUG method, the statistical index (mean value) was directly calculated as the probability distribution of a subgraph for each subgraph pattern in positive and negative samples. Next, based on the theory of the discrimination score function in a certain graph, the discrimination score for each subgraph was calculated and discriminative subgraphs were selected accordingly.

Considering the inherent uncertainty in graphs and the limitations imposed by a certain brain network, this paper introduced uncertain graph theory to construct an uncertain brain network and then used the approximate algorithm (unFEPG) to mine frequent subgraphs within the uncertain brain network. Next, discriminative subgraphs were selected using the statistical index (dfsSI) and the discriminative score function. Finally, the discriminative subgraph features were used for classification. Results show that the MUSE and dfsSI method achieves better classification accuracy than the traditional DUG method. Furthermore, to further prove the efficacy of the proposed method, this paper also compared an uncertain brain network with a certain brain network in a bidirectional manner based on a unified subgraph model. Results showed that under certain sparsity conditions (that is, under certain threshold conditions), the classification performance of the uncertain brain network was better than that of the certain network. In addition, we also evaluated the generalization performance of the classification model constructed by the proposed method using our dataset and an independent validation dataset respectively. We also discuss the number of features, model parameters, and classifier parameters.

## Materials and Methods

### Method Framework

**FIGURE 1:**
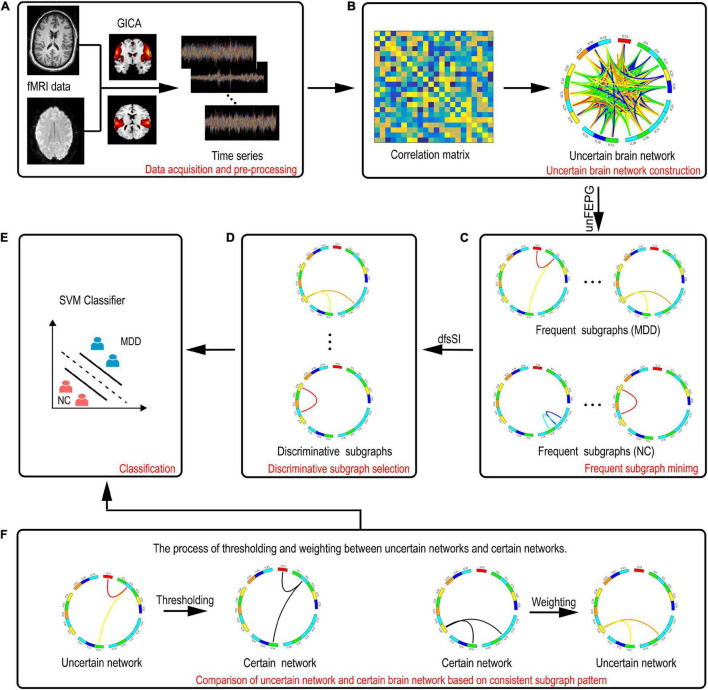
A description of the entire framework for the proposed method. **(A)** Data acquisition and preprocessing. **(B)** Construction of the uncertain brain network. **(C)** Frequent subgraph mining. **(D)** Discriminative subgraph selection. **(E)** Classification. **(F)** Comparison of the uncertain network and certain brain network based on consistent subgraph patterns. The left graph represents thresholding of discriminative subgraphs in the uncertain network. The right graph represents the weighting of discriminative subgraphs in the certain network.

Figure 1 shows the entire flowchart. Specifically, this process focuses on the analysis of uncertain brain network and includes the following parts:

(1) Data acquisition and preprocessing.

(2) Group independent component (IC) analysis.

According to fMRI data, the ICs are estimated.

(3) Construction of uncertain brain networks in which the correlation method is used to construct an uncertain brain network.

(4) Mining frequent subgraphs of uncertain networks using the approximate algorithm method, based on pattern growth of frequent edge, to obtain a frequent subgraph pattern.

(5) Selection of discriminative features utilizing the statistical index and the discrimination score function to obtain discriminative subgraph features.

(6) Support vector machine classification.

A support vector machine (SVM) based on radial basis function (RBF) kernel function is used for classification.

(7) Comparison of the uncertain and certain brain networks.

The uncertain discriminative subgraph is fitted with a threshold and the certain discriminative subgraph is weighted to obtain a consistent subgraph mode. On this basis, the classification performance of the certain and uncertain networks can be compared in a bidirectional manner.

### Data Acquisition and Preprocessing

Following the recommendations of the Shanxi Medical Ethics Committee (reference no. 2012013), all subjects needed to provide their consent to participate. All participants provided written informed consent in accordance with the Declaration of Helsinki, including 38 subjects with first-time, drug-free, major depression disorder (MDD) as the depression group and 28 age and gender-matched healthy volunteers as the normal control (NC) group. All subjects were righthanded. Participants in the depression group participants were first-time, drug-free patients identified by the criteria provided by the American Manual of Diagnostic and Statistical Manual of Mental Disorders, Fourth Edition (DSM-IV) (First and Gibbon, 1997). The severity of depression was determined by the 24 Hamilton rating scale for depression (HAMD) (Williams, 1988) and the clinical global impression of severity (CGI-S) (Guy, 1976). Using a 3T magnetic resonance scanner (Siemens Trio 3-Tesla scanner, Siemens, Erlangen, Germany), resting-state functional magnetic resonance scans were performed on 28 normal and 38 patients with depression. Detailed information relating to the subjects is shown in Table 1. The power analysis for subject inclusion is shown in Supplementary Text S1.

**TABLE 1:** Demographic and clinical characteristics of the subjects.

	NC (*n* = 28)	MDD (*n* = 38)	*P*-value
Age	26.60 ± 9.4 (17–51)	28.40 ± 9.68 (17–49)	0.44*^a^*
Gender (Female/Male)	15/13	23/15	0.57*^b^*
Handedness (Right/Left)	28/0	38/0	–
HAMD	N/A	22.80 ± 13.30 (15–42)	–

*Data are presented as the range (mean ± standard deviation). NC, normal controls; MDD, major depressive disorder; HAMD, Hamilton Depression Rating Scale. ^a^P-value was calculated by two-sample two-tailed t-test; ^b^P-value was computed by two-tailed Pearson’s chi-square test.*

Data acquisition was completed by the First Hospital of Shanxi Medical University and all scans were performed by radiologists who were familiar with the operation of the MRI scanner. All patients underwent complete physical and neurological examinations, standard laboratory tests, and extensive neuropsychological assessments. During the scanning period, subjects were asked to close their eyes, relax, and not to think about anything specific, but to remain awake and not to fall asleep. Scanning parameters were set as follows: 33 axial slices; repetition time (TR) = 2000 ms; echo time (TE) = 30 ms; slice thickness/skip = 4/0 mm; field of view (FOV) = 192 × 192 mm; matrix size = 64 × 64 mm; flip angle = 90°; volumes = 248. Detailed scanning parameters are given in Supplementary Text S2.

Data preprocessing was performed in SPM8 software^1^. First, the dataset was corrected for slice time and head motion. From the final total of 66 subjects, data were not included from any subject with a head movement greater than 3 mm or with rotation greater than 3°. Then, we performed co-registration for spatial correction. Next, images underwent 12-dimensional optimal affine transformation into the standardized Montreal Neurological Institute (MNI) space, using 3 mm voxels. Smoothing was further performed to eliminate the differences between brain structures in different subjects and to improve the signal-to-noise ratio. Linear dimensionality reduction and bandpass filtering (0.01–0.10 Hz) were finally performed to eliminate the effects of line frequency drift and high frequency physiological noise. In addition, we used head, white matter and cerebrospinal fluid signals as covariates for regression analysis to remove nuisance information from images. However, we did not regress global brain signals (Li et al., 2019).

### Group Independent Component Analysis

In the current study, group independent component analysis (GICA) was used to analyze the fMRI data. GICA was carried using the GIFT package^2^. Specifically, the minimum description length (MDL) criterion was applied to estimate the optimal number of decomposition components (Koechlin and Summerfield, 2007) in the normal group and in the depression group. On this basis, we set the final number of ICs to 54. Next, the ICs of each subject was decomposed using the Infomax algorithm, thus resulting in 54 independent spatial components in each subject. The principle of this algorithm was to minimize the mutual information among the components of the output by maximizing the mutual information between the input and the output (Du and Fan, 2013). To strengthen the stability and reliability of the ICs, the Infomax algorithm was run 20 times on ICASSO^3^ by randomly initializing the decomposition matrix; after these repetitions, the same convergence threshold (Nenert et al., 2014) was acquired. Finally, the GICA3 (the third method based on group independent component analysis) algorithm was adopted to reconstruct the data such that the spatial distribution and time series of the ICs of the subjects (Erhardt et al., 2011) could be obtained. See Supplementary Text S3 and Supplementary Table S1 for a detailed explanation relating to the rationality for selecting the 54 ICs.

The ICs extracted by the GICA in this paper not only included the components-of-interest from the brain network but they also included other unrelated components and components with more noise. Therefore, it was necessary to use a prior template matching method to screen out these ICs and to further confirm the components-of-interest using a manual inspection method (Jafri et al., 2008). The screening criteria used for the exclusion of intrinsic connection network components included the following conditions: larger activation areas, where the multiple regression coefficients matched the prior template; the distribution of the main activation regions in the gray matter; the overlap of these regions with known components, such as blood vessels and head movements in low frequency space; and the domination of the power spectrum for the time series in activation regions by low frequency power (Allen et al., 2011). Finally, 32 unrelated or noisy components were removed, and 22 brain network components were retained; these intrinsic connectivity network components were identified as being part of the auditory network, sensorimotor network, visual network, default mode network (DMN), attention network, or frontal lobe network. These 22 brain network components were common regions for the two groups of subjects.

### Construction of the Uncertain Brain Network

#### Uncertain Graph Theory

##### Definition 1 (Uncertain Graphs)

Uncertain graphs are undirected graphs with uncertainties represented as $\tilde{G}$ = (*V*, *E*, *p*) (Khan et al., 2018b; Ke et al., 2020). Of these, *V* = {*v*_1_, *v*_2_, …, *v*_*n*_} refers to the node set, *E*⊆*V*×*V* refers to the probabilistic edge set, and *p*:*E*→(0, 1] is a function denoting the likelihood of the existence of each edge in *E*. That is, *p*(*e*) denotes the probability of the edge about *e* ∈ *E*. A certain graph is a special case of uncertain graph, where the probability of its edges [*p*(*e*)] is 1.

**FIGURE 2:**
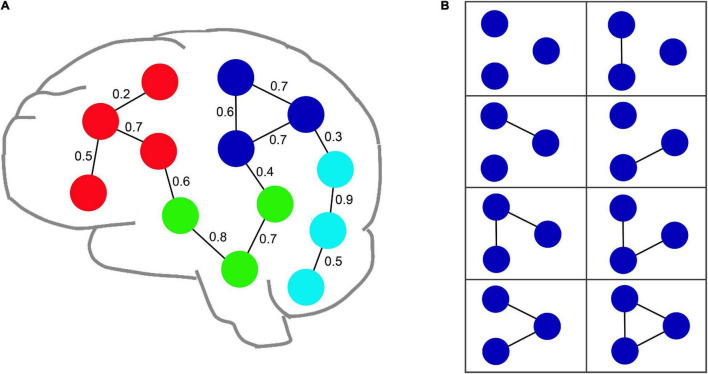
An example of an uncertain fMRI brain network. **(A)** Illustrates an uncertain brain network with thirteen nodes and thirteen edges. The value of edge denotes the probability value of each edge. **(B)** Shows all possible instances for an uncertain graph composed of the blue nodes shown in **(A)**.

An uncertain graph $\tilde{G}$ may include a great quantity of instances, each of which is a certain graph, represented by *G*. Figure 2 shows an example of an fMRI uncertain brain network including thirteen nodes and thirteen edges.

If all edges *E*(*G*) in the graph *G* are extracted from *E*($\tilde{G}$) in terms of the probability *p*(*e*) and *E*(*G*)⊆*E*($\tilde{G}$), then a certain graph *G* = (*V*, *E*) can be implied from an uncertain graph $\tilde{G}$ (denoted as $\tilde{G}$⇒*G*). G is an instance of $\tilde{G}$, and all instances consists of a set *W*($\tilde{G}$) = {*G*|$\tilde{G}$⇒*G*}. The probability that a certain graph *G* ∈ *W*($\tilde{G}$) is implied from an uncertain graph $\tilde{G}$, which is defined by Eq. 1 (Khan et al., 2018b; Ke et al., 2020).


(1) $$\mathrm{Pr}\left[\tilde{G}\Rightarrow G\right]=\prod_{e\in E\left(G\right)}\underset{\tilde{G}}{\mathrm{Pr}}\left(e\right)\prod_{e\in E\left(\tilde{G}\right)-E\left(G\right)}\left(1-\underset{\tilde{G}}{\mathrm{Pr}}\left(e\right)\right)$$

In Eq. 1, *e* refers to the edge of an uncertain graph; *E*(*G*) refers to the edge sets of graph *G*; *E*($\tilde{G}$) refers to the edge sets of graph $\tilde{G}$; ${\text{Pr}}_{\tilde{G}}$(*e*) refers to the existence probability for an edge *e* ∈ *E*($\tilde{G}$).

Notably, the uncertain graph was similar with the weighted graph in terms of its form. However, the largest difference between the two graphs is the understanding of weights. An uncertain graph can be considered as a special edge-weighted graph (Zou et al., 2010) in which the weights refer to the probability of an edge existing between a pair of nodes, thus considering the noisy measurements of the underlying truth. Edge probabilities are semantically different from edge weights, and there is no meaningful way to perform such a casting (Khan et al., 2018b). Moreover, with an uncertainty graph, we can set a threshold probability value and decide to ignore any component with an existence probability below that threshold (Khan et al., 2018b). In recent years, uncertain graphs have been applied to many fields, especially biological networks, mobile *ad hoc* networks, social networks, and other applications where edges are assigned a probability of existence due to a range of factors, such as noisy measurements, the lack of precise information, and inconsistent, incorrect, and potentially ambiguous sources of information (Zhang et al., 2017; Khan et al., 2018a; Li et al., 2020; Saha et al., 2021).

#### Construction of Uncertain Brain Networks

An uncertain brain network is based on uncertain graph theory in which each node represents a region of interest and each edge is associated with a probability *p*(*e*) that relates to the likelihood that a functional connection exists in the brain. In exiting studies, uncertain brain networks were mainly constructed based on Pearson’s correlation method (Kong et al., 2013; Cao et al., 2015a,b; Saha et al., 2021). Therefore, in this paper, we used Pearson’s correlation method to construct an uncertain brain network. Specifically, the locations in the cerebral cortex that corresponded to the remaining 22 ICs (after removing noise components) were used as the nodes of the uncertain brain network. For each subject, a 22 × 22 correlation matrix was obtained based on Pearson’s correlation method; this was calculated by Eq. 2.


(2) $$r_{i,j}=\frac{cov\left(i,j\right)}{\sigma_{i}\sigma_{j}}$$

In Eq. 2, *r*_*i,j*_ denotes the correlation coefficient of the time series relating to the independent component *i* (IC *i*) and independent component *j* (IC *j*). *cov*(*i*, *j*) denotes the covariance of the two independent component time series. σ_*i*_ and σ_*j*_ represent the standard deviations of the time series about the two ICs, respectively.

Given that the edges of the uncertain network were associated with a probability that illustrates the likelihood of whether this edge should exist or not, the correlation matrix was processed according to Eq. 3.


(3) $$b_{ij}=\left\{ \begin{array}{c} r_{ij},r_{ij}\geq 0\\ 0,r_{ij}<0 \end{array}\right.$$

In Eq. 3, *b*_*ij*_ denotes the edge value of IC *i* of IC *j* in the uncertain brain network model (Kong et al., 2013; Saha et al., 2021). Positive correlations were used as edge values (uncertain links) among different brain regions to form uncertain networks (Kong et al., 2013; Cao et al., 2015a; Tokuda et al., 2018).

### Frequent Subgraph Mining of Uncertain Brain Networks

#### Subgraph Theory

##### Definition 2 (Subgraph)

In definition 2 (subgraph), *g* = (*V*′, *E*′) and *G* = (*V*, *E*) denote two certain graphs, separately. If *V*′ ∈ *V*and *E*′ ∈ *E*, then *g* denotes a subgraph of *G*, or *G* contains a subgraph *g* (denoted as *g*⊆*G*) (Kong and Yu, 2014).

Given an uncertain graph, the probability of $\tilde{G}$ containing subgraph g is expressed by Eq. 4.


(4) $$\begin{array}{c} \mathrm{Pr}\left[g\subseteq \tilde{G}\right]=\sum_{e\in E\left(g\right)}\mathrm{Pr}\left(\tilde{G}\Rightarrow G\right)\cdot I\left(g\subseteq G\right)=\\ \left\{ \begin{array}{c} \prod_{e\in E\left(g\right)}p\left(e\right),E\left(g\right)\subseteq E\left(\tilde{G}\right)\\ 0,\mathrm{otherwise} \end{array}\right. \end{array}$$

In Eq. 4, *e* refers to an edge of the uncertain graph; *E*(*g*) refers to all edges in the graph *g*; *E*($\tilde{G}$) refers to all edges in the graph $\tilde{G}$; Pr($\tilde{G}$⇒*G*) have the same meaning as in Eq. 1; when *g*⊆*G*, then *I*(*g*⊆*G*) = 1, if not, then *I*(*g*⊆*G*) = 0; *p*(*e*) represents the probability of the edge about *e*∈*E*(*g*).

##### Definition 3 (Support Degree)

Definition 3 (support degree) assumes that the uncertain graph dataset *W*($\tilde{D}$) including all of the certain graph set *D* is a probability distribution; the support degree of subgraph *g* in the middle is a probability distribution, as defined by Eq. 5.


(5) $$\left[\begin{array}{cccc} g_{1} & g_{2} & \ldots & g_{m}\\ \mathrm{Pr}\left(g_{1}\right) & \mathrm{Pr}\left(g_{2}\right) & \ldots & \mathrm{Pr}\left(g_{m}\right) \end{array}\right]$$

In Eq. 6, the different subgraph patterns of *W*($\tilde{D}$) are *g*_1_, *g*_2_…, *g*_*m*_; $\mathrm{Pr}(g_{k})=\mathrm{Pr}[g_{k}\subseteq \;{\tilde{G}}_{i}](k=1,\text{…},m;i=1,\text{…},n)$ represents the probability of ${\tilde{G}}_{i}$ including subgraph g which can be referred to as the support degree of subgraph *g*_*k*_(Li et al., 2012); *m* refers to the number of subgraph patterns, *n* refers to the number of uncertain graphs; *k* refers to *k*th subgraph patterns; *i* refers to the *i*th uncertain graph. Based on this, the expected support degree of subgraph *g*_*k*_ is defined by Eq. 6.


(6) Esup(gk,D∼)=1n∑i=1nPr[gk⊆G∼]i

In Eq. 6, Pr[gk⊆G∼]i has the same meaning as in Eq. 6. If the *Esup*(*g*_*k*_, $\tilde{D}$) is more than the threshold *minsup*, then the subgraph is regarded as a frequent subgraph.

#### Frequent Subgraph Mining Based on the Pattern Growth of Frequent Edges

Frequent subgraph patterns are an important structural feature of uncertain networks and balance local with global graph topological information (Zou et al., 2009; Kong and Yu, 2014; Yuan et al., 2016; Chen et al., 2019). Considering the limitations of MUSE algorithm, in the present study, we improved the algorithm and proposed an approximate algorithm: a frequent subgraph pattern mining algorithm based on pattern growth of frequent edge in an uncertain network (unFEPG) in which pattern growth of frequent edge was employed to substitute the original pruning process on the frequent subgraph. The specific idea and process used by the algorithm was as follows.

We assumed that the given uncertain graph dataset D∼={G∼,1G∼,2…,G∼}n contained *n* uncertain graphs and that $\tilde{G}$_*i*_ represents the *i*th uncertain graph in $\tilde{D}$. Then, *y* = [*y*_1_, *y*_2_, …, *y*_*n*_]^*T*^ denotes the class labels vector and the class labels are given by *y*_*i*_ ∈ {−1, + 1}. From this, the graph for the depression group in this study can be represented as D∼=MDD{G∼|iG∼∈iD∼∧yi∈+1} while that for the normal group is represented as D∼=NC{G∼|iG∼∈iD∼∧yi∈-1}.

The main concept behind the unFEPG algorithm is to construct a multi-layer sub-search space and select frequent subgraphs from all subgraphs contained in each layer of the sub-search space in all sub-search space. The frequent subgraphs in all sub-search spaces constituted the frequent subgraphs in the MDD group and the NC group. Of these, all subgraphs in each layer sub-search space were obtained using the unFEPG method. The unFEPG algorithm mainly consists of the following steps. Firstly, we took the edges in uncertain brain networks as the subgraphs of the 1-layer search space, calculated the expected support degree (Eq. 7) corresponding to each edge and compared this with the threshold *minsup*. Finally, the edge whose expected support degree was greater than or equal to *minsup* was regarded as a frequent edge and added to the 1-subgraph pattern set in corresponding sub-search space (notably, the frequent edges here were also frequent subgraphs), and the number of frequent edges *k* was set as the number of subgraph search spaces. Secondly, based on the 1-subgraph pattern set, we used the pattern growth of frequent edge method to construct the *i*-layer (*i* = 2,3, …, *k*) sub-search space. Next, we judged all subgraphs in the *i*-layer sub-search space to assess whether they were frequent according to the rules of frequent subgraphs. If the conditions were met, then we defined this as a frequent subgraph and added it to the *i*-subgraph pattern set in corresponding sub-search space. Finally, if the *i*-subgraph pattern set was null or *i* ≥ *k*, then ended the search of sub-search space process and search the next sub-search space. Otherwise, set *i* = *i*+1, iterate (2)-(3). In the next section, each step was described in detail.

The specific steps required to obtain the 1-subgraph pattern set are as follows. Given the input uncertain graph dataset D∼={G∼,1G∼,2…,G∼}n and the threshold *minsup*; then all the subgraph patterns in $\tilde{D}$ constitute the whole search space. First, the edges in the uncertain brain networks were regarded as subgraphs of the 1-layer search space. Then, the expected support degree (Eq. 7) of each edge in the MDD group and the NC group was calculated and compared with the threshold *minsup*. If the expected support degree of the edge was greater than *minsup*, then the edge was denoted as a frequent edge and added to the 1-subgraph pattern set. Note, the frequent edges observed during this step were frequent subgraphs. If the 1-subgraph pattern set contains the edges *m*_1_, *m*_2_, …, *m*_*k*_, then the whole search space can be divided into *k* sub-search spaces that do not intersect each other, where the *i*-subgraph pattern set was distributed in the *i*-layer of the search space. In addition, to reduce the comparison of repeated graphs, we did not include edges with subscripts less than *i* in the *i*-th sub-search space.

The specific steps used to acquire the *i*-subgraph pattern in corresponding sub-search space were as follows. (1) based on the 1-subgraph pattern set, the pattern growth of frequent edge method was adopted to construct the *i*-layer (i = 2,3,…,*k*) sub-search space. (2) The pattern growth of frequent edge method is based on the frequent edges in the 1-subgraph pattern set, each frequent edge is selected to be added to the *i*-1 subgraph pattern set in a retrospective manner. Here, to reduce the comparison of repeated graphs and computation cost, frequent edges were only selected if their subscripts were less than *i* in the 1-layer subgraph pattern set. (3) according to the rules of frequent subgraphs, all subgraphs in the *i*-layer sub-search space were only judged if they are frequent subgraphs. When a subgraph satisfied the conditions required by frequent subgraphs, then it was regarded as a frequent subgraph and added to the *i*-subgraph pattern set in the sub-search space. The specific condition for a subgraph to be a frequent subgraph was that the subgraph must be connected, and its expected support was greater than or equal to *minsup*. (4) the process was terminated if the *i*-subgraph pattern set was null or *i* ≥ *k* and search the next sub-search space. Otherwise, set *i* = *i*+1, iterate (2)-(3). The detailed algorithm for this process is shown in Tables 2, 3. Supplementary Text S4 shows an example to illustrate the unFEPG algorithm. Note that in the *i*-subgraph pattern set, the frequent subgraphs are all *i* edges.

**TABLE 2:** Algorithm for frequent subgraph mining based on frequent edges.

Input: The uncertain graph dataset $\tilde{D}$ and minimum expected support degree *minsup*
**Recursive subgraphs mining:** (1) Traverse $\tilde{D}$ to acquire all 1-layer sub-search space in $\tilde{D}$, and calculate the expected support degree *Esup*(*g*, $\tilde{D}$) according to formular 7. (2) If *Esup*(*g*, $\tilde{D}$)≥*minsup* of the subgraph (frequent edge) in the 1-layer search space, then add it to the 1-subgraph pattern set *M*and the frequent subgraph pattern dataset *R.* (3) Set the number of subgraph sub-search spaces as *k* according to the number of subgraphs in*M.* (4) For each subgraph in *M*, employ the algorithm for pattern growth given in Table 3 to acquire the *i*-layer (*i* = 2,3, …, *k*) corresponding sub-search space *N.* (5) For the subgraph in *i*-layer (i = 2,3, …, *k*) search space*N*, use formular 7 to calculate the expected support *Esup*; similarly, if *Esup*≥*minsup* in the *i*-layer (*i* = 2,3, …, *k*) sub-search space, then add it the *i*-subgraph pattern set in *i*-layer (*i* = 2,3, …, *k*) sub-search space and the frequent subgraph pattern dataset *R.* (6) *i* = *i*+1, repeat steps 4 and 5 until *i*-subgraph pattern set was null or *i* ≥ *k* and search the next sub-search space. **Output:** The frequent subgraph pattern dataset *R*from $\tilde{D}$.

**TABLE 3:** Algorithm for pattern growth.

Input: The (*i*-1)-subgraph pattern set in (*i*-1)-layer (*i* = 2,3, …, *k*) sub-search space and the 1-subgraph pattern set *M.*
**Pattern growth:** (1) Label the sub-search space where the (*i*-1)-subgraph pattern is defined as *i*-1. (2) For each subgraph pattern (frequent edge) in *M*, if it has a label > *i*-1, then add it to the (*i*-1)-subgraph pattern set to acquire the new subgraph *s* (the number of edge in the subgraph is *i*). (3) If subgraph *s* is connected, then add it to the *i*-layer (*i* = 2,3, …, *k*) sub-search space *N.* **Output:** The *i*-layer (*i* = 2,3, …, *k*) sub-search space *N.*

Based on the uncertain brain networks in the MDD group and NC group, we were able to obtain each layer subgraph pattern set (that is, frequent subgraphs in each layer search space). These frequent subgraphs constituted the final frequent subgraphs of the two groups of subjects.

### Discriminative Subgraph Feature Selection for Uncertain Brain Networks

The number of frequent subgraphs extracted by uncertain brain networks was very large. If all frequent subgraphs participated in the classification, then this would reduce the classification performance. Not all frequent subgraphs had discriminative ability; in fact, only a few subgraphs are known to possess discriminative ability (Guo et al., 2017). Thus, it was necessary to select discriminative subgraphs as classification features. In previous studies, researches usually measured the discrimination score for each subgraph to select discriminative subgraphs (Guo et al., 2017, 2018; Cui et al., 2018). The larger the discriminative score, the stronger the discriminative ability of the subgraph. In conventional certain networks, the discrimination scores of the subgraph features were applied into discriminative subgraph mining, in which the edge of each network was certain. On this basis, there is clear certainty relating to the number of times the subgraph feature appears in the network. Accordingly, a discriminative subgraph can be selected according to the discrimination scores (for example, the difference in frequency for which a subgraph features in two groups of subjects) (Guo et al., 2017). However, when the uncertainty of the edges was presented in the form of a graphs (i.e., an uncertain network), a subgraph feature only existed in a graph with a specific probability. Thus, the discrimination scores for a subgraph feature were no longer certain values; rather, they were random variables with probability distributions (Gao and Wang, 2010). Therefore, due to the uncertainty of the edges being taken into account, the selection of discriminative subgraphs in the uncertain brain network was every different from that of a conventional certain network (Kong et al., 2013). Supplementary Figure S1 shows an example to illustrate the differences of discriminative capabilities between subgraphs from uncertain and certain networks.

Considering the problem of low classification accuracy of discriminative subgraphs in existing uncertain brain network research, we combined the calculation method used to define the discriminative score in certain and uncertain networks and proposed a novel discriminative feature selection method based on statistical index (dfsSI) to select discriminative subgraph features from an uncertain brain network.

First, the selection method used for a discriminative subgraph in a certain network was referenced. In a certain network, a discriminative subgraph was obtained by counting the number of times a subgraph appeared in positive and negative samples and then applying this to the discriminative score function to calculate the discriminative score. The higher the discriminative score, the stronger the discriminative ability of the subgraph. As mentioned earlier, a certain network can be regarded as a special uncertain network with a probability of 1 on each edge. On this basis, the number of times a subgraph appears can be regarded as the sum of the probabilities in a positive and negative sample. This was the methodology applied in the current study. In addition, considering the balance between sample sizes, we further introduced the statistical index method for uncertain networks. In other words, the statistical index was introduced to calculate the probability distribution of a subgraph appearing in the two groups of subjects respectively. Then, we applied this into the discriminative score function to calculate a discriminative score for each subgraph.

Many statistical indicators have been used in existing studies, including mean, median, and range (Chen, 2014; Franceschelli et al., 2017; Ben-Aharon et al., 2019). In this study, we adopted the mean index as a statistical index as this has been widely applied to discriminative subgraph mining in uncertain networks (Zou et al., 2009, 2010; Kong et al., 2013). The mathematical definition of the mean values for this study were given as shown in Eqs 7, 8.


(7) $$M\text{ean}\left(g,\overset{∼}{\underset{MDD}{D}}\right)=\frac{1}{M}\sum_{i=1}^{M}\mathrm{Pr}\left[g\subseteq {\tilde{G}}_{i}\right]$$

(8) $$M\text{ean}\left(g,\overset{∼}{\underset{NC}{D}}\right)=\frac{1}{N}\sum_{i=1}^{N}\mathrm{Pr}\left[g\subseteq {\tilde{G}}_{i}\right]$$

In Eqs 7, 8, $\overset{∼}{\underset{MDD}{D}}$ represents the set of uncertain networks for the depression group; $\overset{∼}{\underset{NC}{D}}$ represents the set of uncertain networks for the normal group; ${\tilde{G}}_{i}$ represents the uncertain brain network for the *i*th subject; *g* represents a frequent subgraph; $\sum_{i=1}^{N}\mathrm{Pr}\left[g\subseteq {\tilde{G}}_{i}\right]$ represents the corresponding probability values for subgraph *g* contained in ${\tilde{G}}_{i}$; *M* refers to the number of subjects in the depression group; and *N* refers to the number of subjects in the normal group. After calculating the mean value for frequent subgraphs, we then carried out the discriminative score function to obtain discriminative scores for frequent subgraphs. In uncertain graph theory, the common discriminative score functions contain confidence (Jin and Wang, 2011), frequency ratio (Yan et al., 2008), G-test score (Gao and Wang, 2010), and Hillbert Schmidt independence criterion (HSIC) (Kong et al., 2011). The confidence method possesses good subgraph discrimination ability and a strong generalization ability, which has been widely applied in previous researches (Jackson and Read, 2010a,b). Therefore, in the present study, we used the confidence method as the discriminative score function to select discriminative subgraphs. We measured the confidence values of the frequent subgraphs respectively for the MDD group and the NC group. Then we arranged the two group values in reverse order, and selected the top-*k* values in the two groups as discriminative subgraph features. Finally, we acquired 2*k* discriminative subgraphs. The specific definition was expressed by Eqs 9, 10.


(9) $$Confidence\left(n_{MDD}^{g},n_{NC}^{g}\right)=\frac{n_{MD\text{D}}^{g}}{n_{MDD}^{g}+n_{NC}^{g}}$$

(10) $$Confidence\left(n_{MDD}^{g},n_{NC}^{g}\right)=\frac{n_{\text{NC}}^{g}}{n_{MDD}^{g}+n_{NC}^{g}}$$

In Eqs 10, 11, $n_{MD\text{D}}^{g}$ refers to $Mean(g,\underset{MDD}{\tilde{D}})$; $n_{NC}^{g}$ refers to $Mean(g,\underset{NC}{\tilde{D}})$.

### Classification

The discriminative subgraph was selected using the dfsSI method (that is, the mean value was used as the statistical index value and applied to the discriminative score function to select the discriminative subgraph). Then, the classification model was constructed according to the discriminative subgraph feature. In this study, we adopted a SVM classifier based on the RBF kernel for classification. Here, we used the LIBSVM toolkit in MATLAB to classify our data^4^.

We adopted the 10-fold cross validation to evaluate classification performance. The samples were randomly divided into 10 parts, with one part regarded as the test set and the other nine as the training set. Finally, the average of 10 results was measured to assess the performance of the classifier. In addition, to increase the accuracy of our results, the 10-fold cross-validation was repeated 100 experiments in the experiment, and the average value of the 100 experiments was considered as the final result.

## Results

### Intrinsic Connectivity Network

In this study, we chose 22 ICs using GICA. Supplementary Figure S2 shows the spatial maps of these 22 ICs. In terms of the spatial maps of each IC, the inherently connected network to which they belong was determined, as shown in Supplementary Figure S2.

These 22 ICs were similar to those identified in previous work (Beckmann et al., 2005; Calhoun et al., 2008; Smith et al., 2009; Allen et al., 2011). Here, we described these 22 ICs in detail. Resting-state networks are grouped by their anatomical and functional properties. IC 15 forms a rather prototypical representation of the large parts of the auditory system (AUD), mainly including bilateral activation of the superior temporal gyrus (Seifritz et al., 2002; Specht and Reul, 2006). The Sensorimotor networks (SM) were captured by five components (ICs 4, 8,11, 22, and 36) situated in the vicinity of the central sulcus, mainly including activation of the left precentral gyrus, right postcentral gyrus, bilateral activation of the paracentral lobule, supramarginal gyrus and supplementary motor area (Krienen and Buckner, 2009; Abouelseoud et al., 2010). The visual system (VIS) is also represented by six components (ICs 10, 19, 32, 34, and 38) in good agreement with the anatomical and functional delineations of occipital cortex. The main active regions were the lingual gyrus, cuneiform lobe, suboccipital gyrus, talus gyrus and middle temporal gyrus (Grill-Spector and Malach, 2004). The DMN was captured by three independent components (ICs 16, 18, and 31); the main active regions were located in the precuneus lobe, lingual gyrus and temporal lobe etc. The attention network (ATTN) was captured by six independent components (ICs 24, 25, 30, 35 39, and 40); the main active regions were located in the frontal lobe, parietal lobe, precuneus lobe, temporal lobe and angular gyrus (Corbetta and Shulman, 2002; Vincent et al., 2008). Finally, frontal networks (FRONT; ICs 33 and 43) known to mediate executive as well as memory and language functions was observed, whose active regions were located in the medial prefrontal cortex and parietal lobe (Koechlin et al., 2003; Koechlin and Summerfield, 2007).

### Frequent Subgraph Patterns and Discriminative Subgraph Patterns

After constructing the uncertain brain network, the unFEPG algorithm was separately used to mine the frequent subgraphs from the NC and MDD groups. When the *minsup* parameter was set to 0.25, 289 frequent subgraphs were mined from the NC group and 192 from the MDD group. Specific information relating to the frequent subgraphs is given in Supplementary Table S2.

**FIGURE 3:**
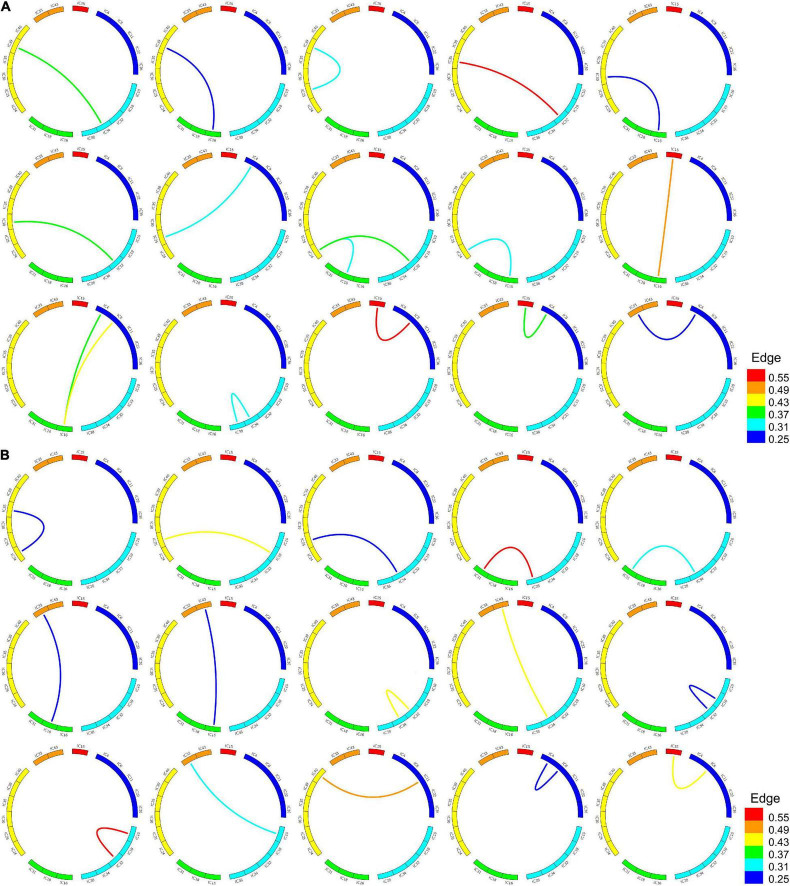
Frequent subgraphs of MDD and NC group. **(A)** Represent top 15 discriminative subgraphs in MDD group. Edge refers to the edges are assigned with a probability of existence in MDD group. **(B)** Represent top 15 discriminative subgraphs in NC group. Edge refers to the edges are assigned with a probability of existence in NC group.

**FIGURE 4:**
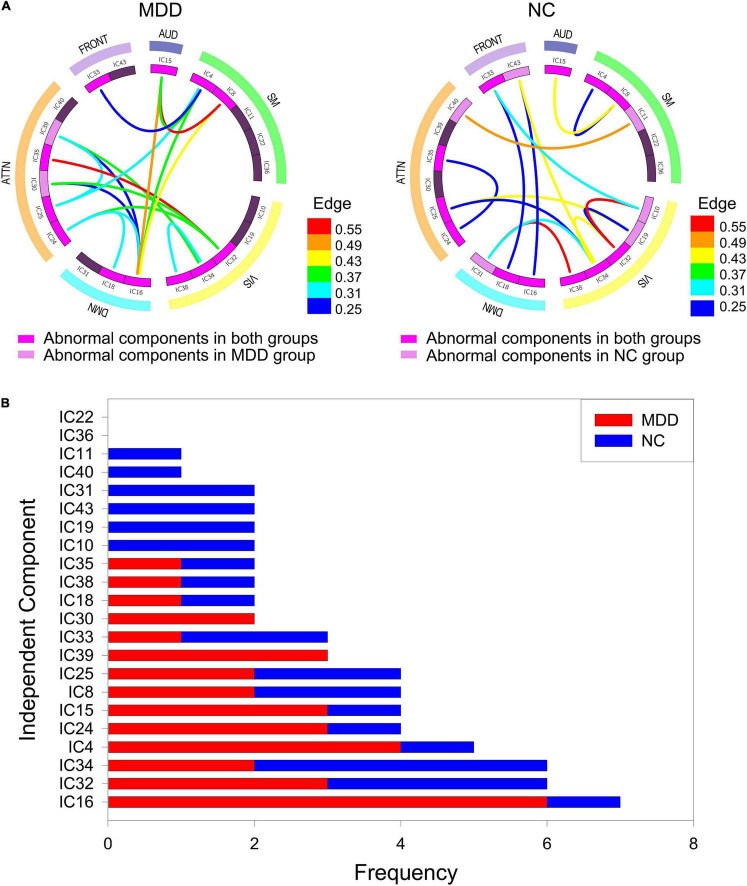
The abnormal independent components of subgraph feature. **(A)** Represents all discriminative subgraphs were combined in each group. AUD, auditory network; SM, sensorimotor network; VIS, visual network; DMN, default mode network; ATTN, attentional network; FRONT, frontal network. Edge refers to the edges are assigned with a probability of existence. **(B)** Represents a statistical chart about the occurrences of these independent components in **(A)**.

According to the frequent subgraphs, the dfsSI algorithm was used to calculate discriminative scores for the frequent subgraphs. Then, discriminative subgraphs from the NC group and MDD group were selected based on discriminative scores. To ensure a balanced number of subgraph features, we respectively selected the top 15 frequent subgraphs with the highest discriminative scores from the two groups of subjects as the discriminative subgraph features to perform classification, as shown in Figure 3 (see Section “The Influence of the Number of Features” for a discussion of the number of subgraph features). To analyze the difference of the discriminative subgraphs between the two groups, we combined 15 discriminative subgraphs from each group, as shown in Figure 4A. Results showed that the abnormal components obtained by the two sets of discriminative subgraphs were almost identical, and included IC16, IC32, IC34, IC4, IC8, IC15, IC24, IC25, IC33, IC18, IC38, and IC35. On this basis, we counted the number of times each IC appeared in all discriminative subgraphs to select the most discriminative components for MDD, as shown in Figure 4B. The results showed that the top 3 abnormal components were IC16, IC32, and IC34. Of these, IC16 occurred the most frequently in the abnormal components (seven times). This was followed by IC32 and IC34 respectively (occurring six times each).

### Classification Results

Based on the discriminative subgraph features, we next assessed classification performance by calculating classification accuracy, sensitivity, and specificity, and the area under the curve (ROC).

We evaluated classification performance based on probability values representing functional connections (PV-FC), the unFEPG method and by combining the unFEPG method with the dfsSI method; then, we compared these two outcomes with the traditional DUG method. First, the DUG method applied Pearson’s correlation method to construct an uncertain brain network. Secondly, the probability distribution for each current subgraph was calculated based on dynamic programming, in which a current subgraph was selected based on a DFS-code tree in gSpan. Then, based on the probability distribution and the values obtained by discriminant score function (confidence) for each current subgraph, statistical indicator (discriminative scores) was acquired. Furthermore, we set the minimum expected frequency (*min_sup*) and the minimum discriminative score (θ), and then compared the expected frequency and discriminative scores for each current subgraph with *min_sup* (*min_sup* was set as 0.25) and θ. If these values were greater than *min_sup* and θ, then the current subgraph was added to the discriminative subgraph set. Otherwise, the sub-tree of the current subgraph was pruned by the branch-and-bound algorithm. Next, a recursion process based on a depth-first search was carried out to identify other discriminative subgraphs. Finally, the top 15 discriminative subgraphs were selected as subgraph features for classification. The classification results for these methods are summarized in Table 4. We found that the accuracy of the unFEPG method, when combined with the dfsSI method, reached 92.9%; this was higher than other three methods (PV-FC, the unFEPG method and the traditional DUG method).

**TABLE 4:** Comparison of classification performance for different researches.

Method	Research	Disease	Accuracy	Sensitivity	Specificity
Frequent subgraph mining of uncertain graphs	PV-FC	MDD	77.85%	81.18%	72.43%
	unFEPG method	MDD	79.15%	86.58%	65.29%
	unFEPG and dfsSI method	MDD	92.90%	93.40%	85.83%
	DUG method (Kong et al., 2013)	ADNI	71.70%	–	–
		MDD	81.04%	88.50%	68.26%

*The PV-FC method represents probability values representing functional connections. The unFEPG method represents frequent subgraph pattern mining algorithm based on pattern growth of frequent edge. The unFEPG and dfsSI method represents combining frequent subgraph pattern mining algorithm based on pattern growth of frequent edge and discriminative feature selection method based on statistical index. The DUG method represents the traditional discriminative feature selection for uncertain graph classification algorithm. MDD, major depressive disorder; ADNI, Alzheimer’s disease.*

## Comparison of Uncertain and Certain Brain Networks

Considering inconsistency between uncertain and certain graphs with regards to subgraph features, and their different forms of feature characteristics, we used consistent discriminative subgraph patterns to bidirectionally compare the classification performance of uncertain networks and certain networks.

### Thresholding Discriminative Subgraphs in Uncertain Brain Networks

**FIGURE 5:**
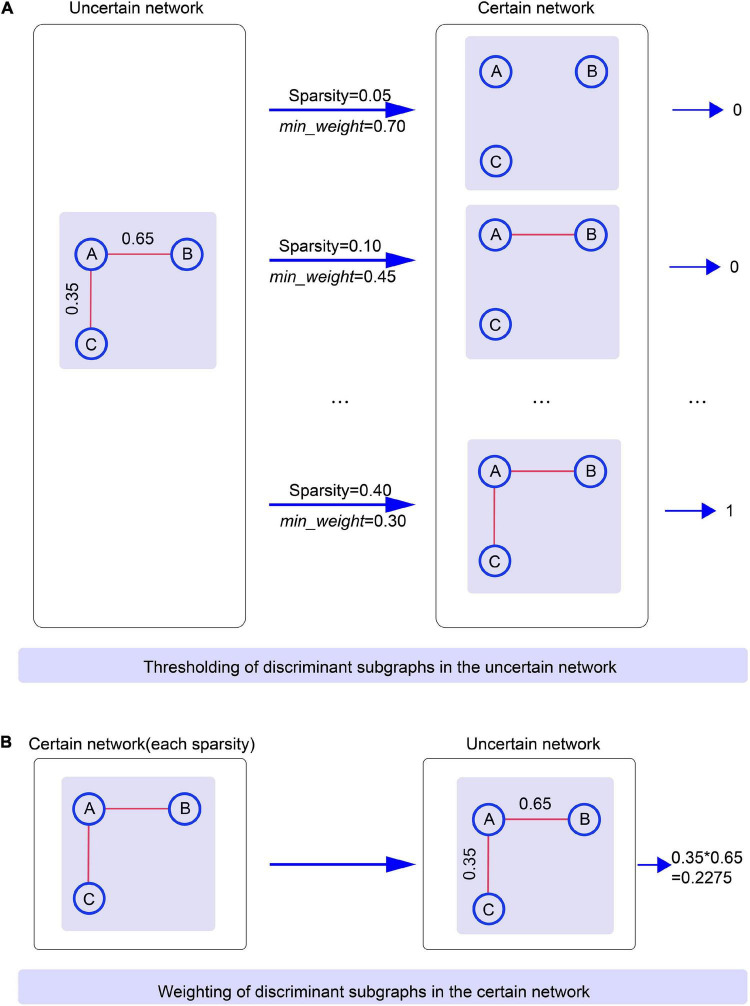
The process of thresholding and weighting between uncertain networks and certain brain networks. Specific clarification is that **(A)** Illustrates the thresholding of discriminant subgraphs in the uncertain network. **(B)** Illustrates the weighting of discriminant subgraphs in the certain network.

To assure that the subgraph patterns were consistent when comparing the classification performance of uncertain and certain networks, we first carried the discriminative subgraph acquired from uncertain brain networks as subgraph features, and then utilized the thresholding method to map them to the certain network. The specific thresholding method process is as follows. First, the probability values for all edges in the uncertain brain network were ordered in reverse order. Then, based on the selected sparsity, the minimum weight at which an edge can exist was regarded as *min_weight*. When the value of an edge of a subgraph in the uncertain network was larger than *min_weight*, then the edge existed in a certain network, and *vice versa*. Accordingly, we acquired the discriminative subgraph patters for the corresponding certain network. Here, note that we obtained distinct subgraph features for a certain network if the sparsity was set distinctly, and the mapped subgraph pattern for a certain network was not necessarily exited. When the mapped subgraph feature was exitent in the certain network, this was represented as 1 (and 0 it not exitent). Using this method, we were able to construct a classification feature matrix for a certain graph. Figure 5 shows an example of thresholding.

The detailed steps taken to perform thresholding for discriminative subgraphs in an uncertain brain network were as follows. First, after construction of the uncertain brain network, we separately used the unFEPG algorithm to the NC group and then to the MDD group to obtain corresponding frequent subgraphs. Next, we used the dfsSI method to measure discriminative scores and extracted the top *k* subgraph features from the NC group and the MDD group as discriminative subgraph features; ranging features were set to 10–130 with a step size of 10. Then, based on the specific sparsity in a certain brain network, and by applying the thresholding method, the discriminative subgraph features in the uncertain network were changed into the corresponding discriminative subgraph in the certain network. Accordingly, we were able to construct a corresponding classification feature matrix for the certain brain network. Finally, SVMs were adopted to carry out classification and the 10-fold cross-validation was repeated 100 experiments to validate the classification performance.

### Weighting of Discriminative Subgraphs in the Certain Brain Network

In this part of the study, we used the well-known gSpan algorithm (Yan, 2002) to extract frequent subgraphs from the certain network. Due its high efficiency for graph traversal and subgraph mining, the gSpan algorithm has been widely employed in neuroimaging (Du et al., 2016; see Supplementary Text S5). To ensure the consistency of this experiment, the maximum total number of discrimination subgraphs for the certain network was set at 130.

Next, we first took the discriminative subgraph patterns obtained from certain networks as subgraph features, and then proposed the weighting method to map them to the uncertain network. The specific weighting method process was as follows. The weight of each edge in the certain network was separated into two values: 0 and 1; in other words, the edge of certain network includes two states, existent and non-existent. During the procedure of subgraph conversion, each edge weight in the certain network was regarded as the probability of the edge in the uncertain network. Here, it should be noted that according to the specific sparsity, the discriminative subgraph features of each certain network must include a corresponding uncertain discriminative subgraph. An example of weighting is shown in Figure 5.

The detailed steps used to weight discriminative subgraphs in the certain brain network were as follows. First, a corresponding certain network was constructed by ranging different sparsity from 0.05 to 0.4, with a step size of 0.05. Second, based on each brain network being constructed with a specific sparsity, the gSpan algorithm was used to mine frequent subgraphs. Third, the discriminative score was calculated using the frequency differences for the NC group and MDD group. The top *k* subgraph features for the NC group and the MDD group were then extracted as discriminative subgraph features for the certain brain network; ranging features were set to 10–130 with a step size of 10. Then, for each specific sparsity, based on the weighting method, the discriminative subgraph features in the certain brain network constructed by the specific sparsity were mapped into the corresponding discriminative subgraph in the uncertain network. On this basis, we were able to construct a corresponding classification feature matrix for the uncertain brain network, based on the certain brain network constructed by each specific sparsity. Finally, SVMs were adopted to carry out classification and 10-fold cross-validation was repeated 100 experiments to validate classification performance.

### Comparison of Classification Results

#### Thresholding Discriminative Subgraphs in the Uncertain Brain Network

**FIGURE 6:**
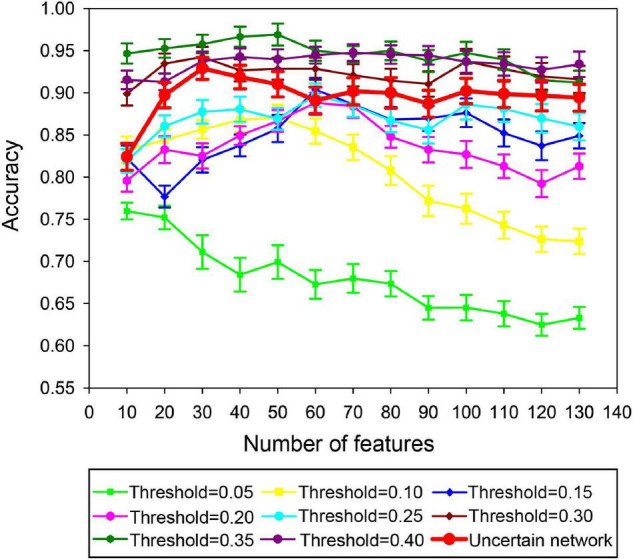
Classification performance using the thresholding method to map discriminative subgraph features of the uncertain brain network to the certain network. The ordinate denotes the accuracy, and the abscissa indicates different feature numbers.

Based on the sparsity in the certain brain network, we used the thresholding method to map discriminative subgraph features of the uncertain brain network to the certain network. Then, under these consistent discriminative subgraph patterns, classification performance was compared between the uncertain network and the certain brain network. Classification results are shown in Figure 6; following the thresholding of discriminative subgraphs for the uncertain network and when considering all discriminative subgraph features, the classification accuracy for the uncertain brain network was better than that of the certain brain network with a sparsity of 0.05–0.25 and was lower than that of the certain brain network with a sparsity of 0.3–0.4.

#### Weighting Subgraphs in the Certain Brain Network

**FIGURE 7:**
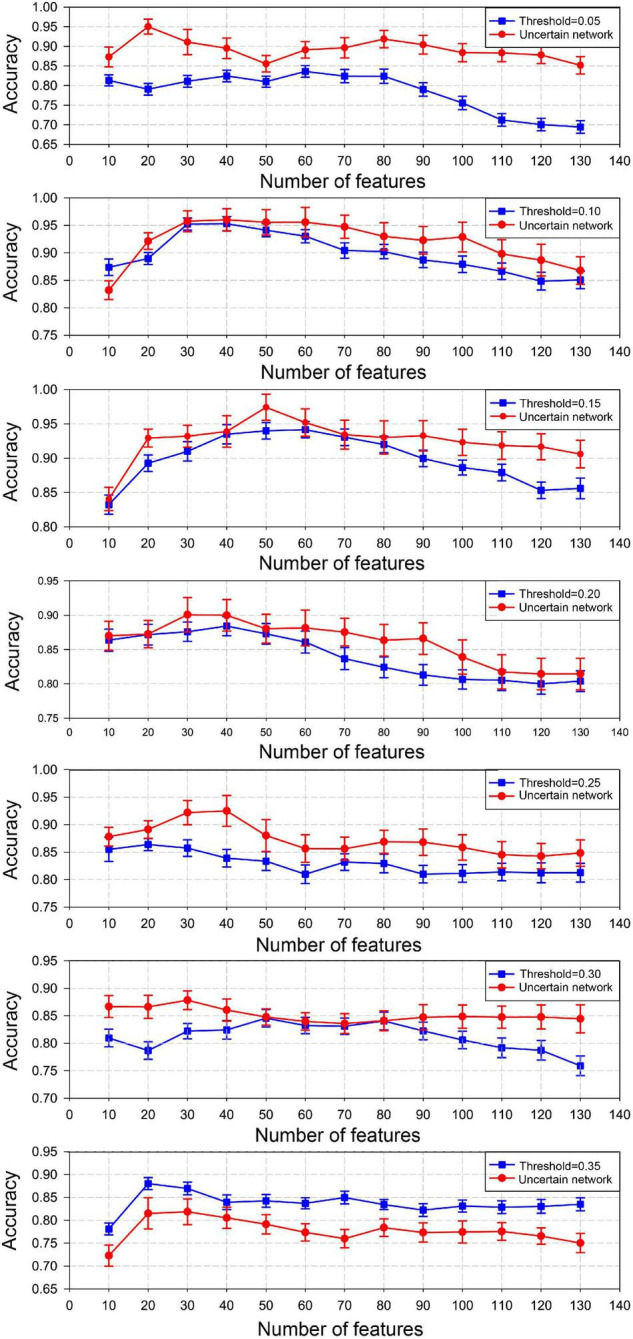
Classification performance using the weighting method to map discriminative subgraph features of the certain brain network to the uncertain network. The ordinate denotes the accuracy, and the abscissa indicates different feature numbers.

Based on the weighting method, the discriminative subgraphs obtained from the certain brain network constructed by each specific sparsity were matched to the uncertain brain network. Then, under these consistent and discriminative subgraph patterns, we compared the classification performance between the certain network and the uncertain brain network. The classification results are shown in Figure 7. With increasing sparsity, the classification accuracy of the uncertain network was consistently higher than that of the certain network. The classification accuracy of the uncertain network was consistently lower than that of the certain network until the sparsity reached 0.35.

## Discussion

Considering the inability to provide effective classification information in the existing subgraph mining and selection methods of uncertain brain network (Papapetrou et al., 2011; Kong et al., 2013), we proposed unFEPG and dfsSI algorithm for subgraph mining and selection in uncertain network. First, we constructed an uncertain brain network to represent the uncertain information with regards to functional connection. Then, the unFEPG algorithm was used to mine frequent subgraphs. Next, dfsSI algorithm was used to select the discriminant subgraph. Finally, SVM was used for classification. The results show that compared with the conventional methods, our uncertain brain network classification method greatly improved the diagnostic accuracy for depression’s disease.

### Abnormal Components

The best classification performance was obtained when 30 frequent subgraph patterns were selected as discriminative subgraph patterns (NC: 15; MDD: 15). Therefore, we analyzed the most discriminative abnormal components obtained by 30 discriminative subgraphs. First, the number of times each IC appeared in all discriminative subgraphs was determined. Then, the top three components were considered to be the most discriminative components (IC16, IC32, and IC34). Of these, IC16 was contained in the DMN. The DMN can be regarded as a high-level cognitive network system; the main function of this network is self-reference. In previous studies, researchers confirmed that the default network was significantly associated with depression (Chen et al., 2015; Zhou H.-X. et al., 2020). In addition, the remaining two discriminative components, IC32 and IC34, were contained in the visual network. The visual network is mainly responsible for the preliminary information processing of stimuli and is regulated by specific regions, such as attention. Existing studies have shown that the pathological mechanisms underlying MDD are related to the visual network; when the visual processing time was significantly increased, the connection pattern was abnormal (Wang et al., 2019). These abnormalities may relate to the selective attention and working memory disorders that occur in depressive patients (Moreno-Ortega et al., 2019). Therefore, the abnormal component results obtained in this experiment are consistent with those in the literature. In addition, we further discussed the pathological mechanism of depression from the brain regions to which the discriminative ICs belong (see Supplementary Text S6). According to brain regions, it could also be concluded that the markers of depression in current study were the same as the existing research.

### Classification Results

The PV-FC, the unFEPG method, the combined unFEPG and dfsSI method, and the traditional DUG method, were respectively applied to the MDD and NC groups for classification purposes, as shown in Table 4. The classification results of the method proposed in this paper (a combination of the unFEPG and dfsSI methods) were higher than those of the PV-FC, the unFEPG and traditional DUG methods. Among the other three methods, PV-FC has the lowest accuracy. This suggested that the classification performance can be improved after using graph theory to measure and characterize the uncertain brain network. Conversely, the classification results obtained by the unFEPG and dfsSI method were higher than those obtained from the unFEPG method. This may be due to the selection of the most discriminative subgraph features on the basis of frequent subgraphs. However, the unFEPG method only utilized frequent subgraph feature mining and did not select discriminative subgraphs. This made led to the inclusion of more features with too much redundant information, fewer features related to class labels, and significant information loss. Accordingly, the generalization ability of the model was reduced (Nouinou et al., 2018). This result was also confirmed by the classification results relating to the selection of the number of discriminative subgraph features (see section “Classification Results”). In the current study, we considered the influence of the number of discriminative subgraph features to evaluate the classification model; the number of discriminative subgraph features ranged from 10 to 100, with a step size of 10. We found that when the number of discriminative subgraph features exceeded 30 and gradually increased, the classification results gradually decreased. This result suggests that some frequent subgraph features were not strongly correlated with brain diseases and could not effectively classify brain diseases (i.e., MDD). Therefore, it is necessary to select more discriminative subgraph features to perform classification when using frequent subgraphs.

The classification results obtained by the unFEPG and dfsSI method was higher than the traditional DUG method. This may be because the unFEPG and dfsSI method fully considered uncertain information in the uncertain brain network. The DUG method was predominantly based on the number of occurrences for each subgraph feature and then used dynamic programming to calculate the probability distribution of all possible occurrences for each subgraph in all samples. For example, for a selected subgraph, the number of possible occurrences of the subgraph in all uncertain brain networks was set as 0-*n* (*n* is the number of subjects). Next, the dynamic programming method was used to calculate the probability distribution of the subgraph in which the number of occurrences of the subgraph was *i* (*i* = 0*, …,n*). Furthermore, the score of the subgraph for when the number of occurrences of the subgraph was *i* was calculated based on the discriminant score function theory in the certain brain network. Finally, based on the probability distribution of all possible occurrences and the corresponding scores, the discriminant score of the subgraph was calculated by using statistical indicators.

However, the unFEPG and dfsSI method did not consider the number of possible occurrences of the subgraph feature in all sample sets, calculate the probability distribution for all possible occurrences, and then determine the discriminant score of a subgraph by measuring statistical indicators. Instead, our method was inspired by a certain brain network that can be regarded as a special uncertain brain network with a probability of 1 for each edge. From the perspective of probability, that is, starting from the uncertain information contained in the uncertain brain network, the number of occurrences of the subgraphs in the discriminant score function was regarded as the sum of the probabilities in all samples. Furthermore, considering the balance between sample sizes, the sum of the probabilities of a subgraph was transformed into a mean probability which was then applied to the discriminant score function to calculate the discriminant score of a subgraph. That is, the uncertain information contained in the uncertain brain network was fully considered. Moreover, compared with the DUG method, the time consumption associated with our combined method was greatly reduced. This result implies that more effective discriminative subgraph features in the uncertain brain network would be selected, the ability to distinguish differences between the MDD and NC groups would be improved, and more accurate biological markers of depression would be obtained when the uncertain information of the uncertain brain network was considered.

Furthermore, we used thresholding and weighting methods to generate consistent discriminative subgraph patterns for uncertain networks and certain networks, and bidirectionally compared the classification performance of these network models. We found that the classification performance of the uncertain network was superior to that of the certain network within a defined sparsity range (Figures 6, 7), regardless of the thresholding method (discriminative subgraphs from the uncertain brain network were converted to the certain brain network) or weighting method (discriminative subgraphs of the certain brain network were converted to the uncertain brain network). The underlying reason is that the number of edges, and the information contained in the certain network, also increased when the sparsity gradually increased. The frequent subgraph pattern of the certain network might be superior to the subgraph pattern of the corresponding uncertain network; thus, the classification accuracy of the certain network was greater than that of the uncertain network.

These results show that the classification accuracy for brain diseases was related to the effective information contained within its subgraph features. To achieve a better classification performance, it is necessary to select a certain brain network with a higher threshold or an uncertain brain network model. Moreover, if an uncertain brain network model is selected, then it is necessary to make full use of the uncertain information related to its functional connections.

### The Discussion of Time Complexity Between This Algorithm and Mining Uncertain Subgraph Patterns Algorithm

Previous studies mainly used three methods for the data-driven analysis of uncertain graphs, including frequent subgraph pattern mining, clustering algorithm calculation for uncertain graphs, and shortest and minimum generation based trees (Potamias et al., 2010; Khan et al., 2018b). The frequent subgraph pattern mining has been used in the field of neuroimaging. Therefore, we proposed to use this novel approximate frequent subgraph algorithm in the current study based on the fact that it has been widely used to apply the frequent subgraph algorithm (the MUSE algorithm; Zou et al., 2009; Kong et al., 2013) on uncertain graphs.

Although the traditional MUSE algorithm adopts the approximation algorithm, alongside expected support and spatial clipping technology, to reduce temporal and space complexity, the computational consumption incurred by this technique is still large (Papapetrou et al., 2011). Therefore, we improved upon this algorithm and proposed an approximation algorithm to generate the unFEPG algorithm, in which the pattern growth of frequent edge was applied to replace the original pruning process on frequent subgraphs. This technique reduced the time complexity associated with the algorithm, thus improving upon the traditional method which takes too long because it considers too many subgraph patterns during frequent subgraph mining. Specifically, the traditional algorithm uses the APRIOR property to crop the entire search space. In contrast, in our research, we adopted the pattern growth method; that is, we replaced the traditional pruning process with the growth of frequent edges on the frequent subgraph, thus reducing time consumption. In addition, the traditional algorithm incorporated the subgraph isomorphism algorithm when calculating the expected support, although the judgment required by subgraph isomorphism is still time-consuming (Huan et al., 2003). However, the unFEPG algorithm proposed in this study encoded edges and applied the depth-first search method, so that we were able to prune the search space within the database. This allowed for additional optimization due to early termination and efficient scheduling to avert expensive subgraph isomorphism tests.

**FIGURE 8:**
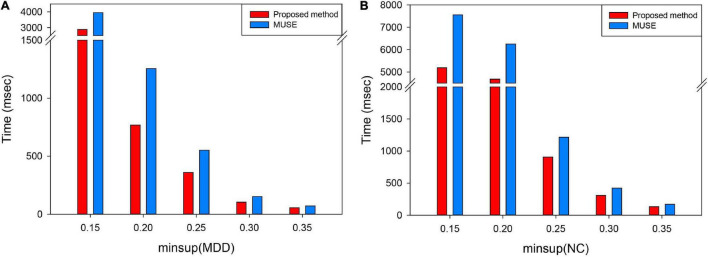
Time complexity under each *minsup* in two groups of subjects. **(A)** Refers to the execution time mining frequent subgraph under each *minsup* in MDD group. **(B)** Refers to the execution time mining frequent subgraph under each *minsup* in NC group. MUSE represents traditional the mining uncertain subgraph patterns algorithm. The proposed method represents unFEPG and dfsSI methods. “msec” refers to millisecond.

In conclusion, this proposed algorithm was superior to the traditional MUSE algorithm in terms of computational consumption. The computational cost for the two algorithms was investigated in each minimum support threshold (*minsup*) using the same dataset. Same as this article, *minsup* was selected from 0.15 to 0.35, with a step size of 0.05; results are shown in Figure 8.

### The Validation of Generalization Performance for the Classification Results

We verified the generalization performance of the proposed method from two aspects. On the one hand, we divided our datasets into a training set and a validation set (they are the same site), where the validation set did not participate in the construction of the classification model at all and did not participate in the process of subgraph feature extraction and selection, but was used directly to validate classification model. On the other hand, we introduced independent validation datasets from other sites and used them to evaluate the generalization performance of classification models.

We randomly divided our dataset into training set and validation set with a ratio of 7:3. As for the training set, after these processes of network construction, subgraph mining, and the selection of discriminative subgraphs, we used the 10-fold cross-validation method to obtain multiple SVM classification models. The generalization performance of the classification model was then evaluated using the validation set. Specifically, the training set data was randomly divided into 10 equal parts, one of which was used as the validation set (Sn) and the remainder as the training set (S-n). S-n was then divided into two parts (training set TR and test set TE). Since different SVM parameter settings led to different results, based on training set TR, classifiers were constructed by choosing different parameters (c, g) values, and the (c, g) value that gave the highest classification accuracy regarding training set TR was determined to be the best parameter. Here, similar to manuscript (c, g) value was set in the [−5, 5] range with a step size of 1. In this way, ten different classification models were built. Then, we used each classification models to predict validation dataset. Finally, the accuracy of each model was averaged as final classification accuracy in this cross-validation. Furthermore, to increase the robustness of our results, dataset partitioning was repeated 20 times and the 10-fold cross-validation in training dataset was repeated 100 times in the experiment, and the mean of the 20*100 results was taken as the final test result. The results are shown in Supplementary Text S7, indicating that under each method, the difference between the test accuracy and the classification results obtained in Table 4 of this paper, about 2–5%, except the subgraph feature with sparsity 0.5. The method proposed in this paper differed by 3%, and finally achieved a test accuracy of 89.56%, which shows that the method proposed in this paper could obtain a satisfactory generalization performance in our dataset.

In addition, we used all site and each site dataset as independent validation datasets to verify the generalization performance of the classifier constructed in this paper. The dataset is obtained from DecNef Project Brain Data Repository^5^. See Tanaka et al. (2021) for the specific demographic information of the subjects. Similar to the validation of above generalization performance. We mainly applied separately the datasets of each site and all site into each of classifiers to perform prediction. The classification results are shown in Supplementary Text S7. The results show that the classifier constructed in this paper has reached more than 70% on all independent data sets, and the accuracy in the HUH dataset was the highest, reaching 75%, which is higher than the results in the existing research (Yamashita et al., 2020). This also indicated that the features obtained by the proposed method can construct an effective MDD classifier. For a detailed discussion, see Supplementary Text S7.

## Methodology

Many parameters were considered in this study. We found that the final classification performance was different when the parameter selection was different. These parameters mainly referred to the feature number, the support degree *minsup* for frequent subgraph mining, the penalty factor *c* in the SVM model, and the kernel parameter *g* in the kernel function. In the next section, we discuss each of these parameters individually.

### The Influence of the Number of Features

**FIGURE 9:**
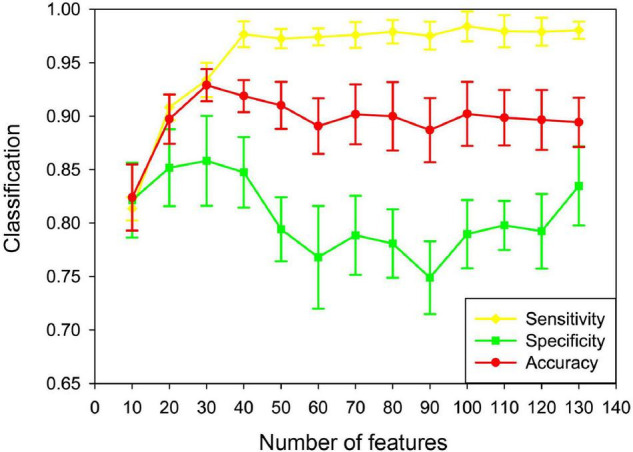
Classification performance based on different feature numbers in the uncertain brain network. Yellow denotes the sensitivity in different feature numbers. Green denotes the specificity in different feature numbers. Red denotes the accuracy in different feature numbers.

In this paper, the unFEPG method was used to obtain the frequent subgraphs of the uncertain brain network, and the dfsSI method was used to calculate the discriminant scores and sort them to select the frequent subgraphs corresponding to the top-*k* discriminant scores as the discriminant subgraphs for classification. Here, the selection of the *k* value will affect the classification, that is, the number of discriminative subgraph features was different, and the classification was different. Therefore, in present study, the number of features was set to 10–130 with a step size of 10. The classification model was respectively constructed and the effect of the number of features on the classification performance was analyzed. It should be noted that when the number of features was larger than 130, the discriminant score value was almost similar or even smaller. This illustrated that the discriminative ability of these subgraphs was not too great. Thus, in present study, the maximum feature number of the discriminative subgraph was set at 130. As is shown in Figure 9, the results show that as the number of features increased, the classification performance gradually decrease after the initial increase. When the number of features was 30, the highest classification accuracy is achieved. The potential reason is that if the feature number is too small, the difference between the MDD group and the NC group is not well expressed; on the contrary, if the number of features is too large, the redundant features would be included, so that affect the construction of the classifier.

### The Influence of the *min_sup* of unFEPG Method

**FIGURE 10:**
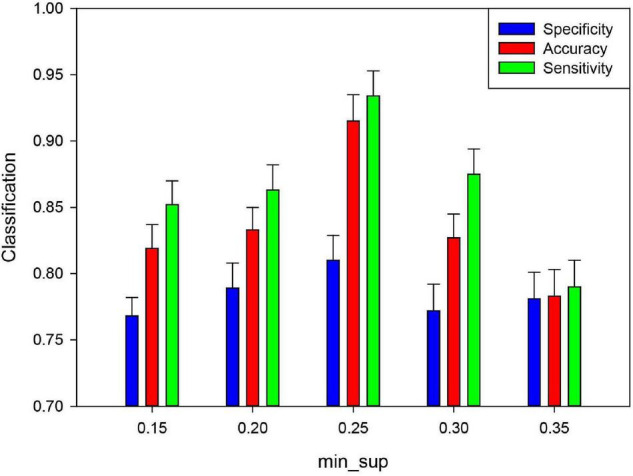
Effect of the minimum support degree (*minsup*) of unFEPG method on the classification performance. Green denotes the sensitivity in different feature numbers. Blue denotes the specificity in different feature numbers. Red denotes the accuracy in different feature numbers.

Based on fMRI data, mining frequent subgraphs from uncertain networks includes the minimum expected support degree (*min*_*sup*), which affects the number of frequent subgraphs mined from the uncertain network. In present study, the *min*_*sup* was set to 0.05–0.35 with a step size of 0.05. These *min*_*sup* was chosen to analyze the classification performance and the other parameters being fixed. Figure 10 show that the classification result was the highest when the *min*_*sup* was set to 0.25. The potential reason is when the *min*_*sup* selected is too large, many effective frequent subgraph features may be missed at the mining stage, which caused the classification performance is lower. When the *min*_*sup* selected is too small, the sizes of the frequent subgraphs will be too large, which caused the redundancy of discriminative subgraph features. This also affected the classification performance. The result indicated that if we want to obtain effective frequent subgraphs, the *min*_*sup* setting should be moderate.

### The Influence of Support Vector Machine Classification Parameters *c* and *g*

**FIGURE 11:**
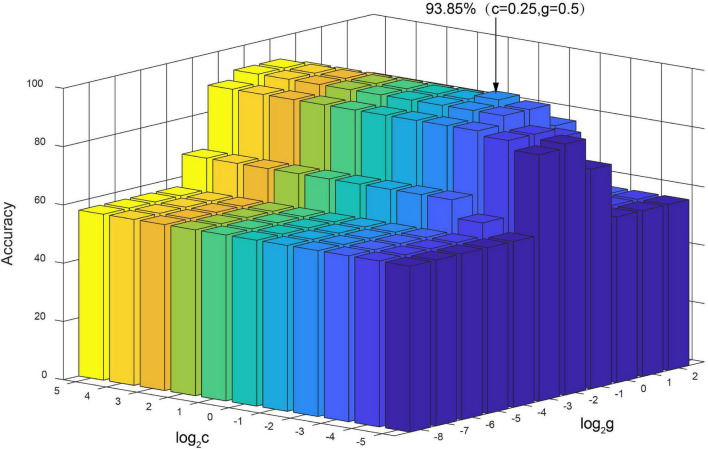
Training classification accuracy of different SVM parameters (*c*, *g*).

In the classification process, the two parameters of the SVM model, the penalty factor *c* and the kernel parameter *g*, strongly effect the classification, and thus it is important to finding the optimal values (Chapelle et al., 2002). The penalty factor *c* is applied to adjust the range of confidence intervals in data subspace. The kernel parameter *g* of the RBF is involved to decide the function for mapping data to a high-dimensional feature space. Selecting the optimal (*c*, *g*) can improve the construction of classification model. For given values of (*c*, *g*), we utilized the K-fold cross-validation method to obtain the training set validation accuracy. The values of (*c*, *g*) that generated the highest validation classification accuracy were selected as the optimum parameters. The ranges of parameter settings applied for *c* and *g* were [2^–5^, 2^5^] and [2^–8^, 2^2^], with a step of 1. Figure 11 displays the results of parameter optimization of (*c*, *g*) when using classification features as training sets. The results show that when *c* = 0.25 and *g* = 0.5, the classification accuracy of the training sets was the highest, reaching 93.85%.

### Limitation

We must also note some limitations of our new method in that the frequent subgraph mining proposed in this paper was a simplified approximate algorithm. This greatly reduced the running time of the algorithm but may have led to the omission of some frequent subgraphs. Therefore, future research should focus on how to further optimize the frequent subgraph mining algorithm for uncertain networks without increasing its computation time. In addition, at the network construction level, we constructed a resting state uncertain brain network in a static form. However, increasing evidence suggests that even in the resting state, the neural activity in the brain still exhibits transient and subtle dynamics. Moreover, these dynamic changes are essential for understanding the basic characteristics relating to brain organization and may be significantly correlated with the pathological mechanisms underlying brain diseases; consequently, these changes may provide useful information for disease classification (Kudela et al., 2017; Zhao et al., 2020). Therefore, dynamic uncertain brain networks could be introduced for the diagnosis of brain diseases and the exploration of pathological mechanisms in future studies. At the feature extraction level, we adopted subgraph features to represent the topology information of uncertain brain networks, which ignore the local topological property information of uncertain brain networks. In future studies, researchers can combine the local properties of uncertain brain networks [e.g., betweenness centrality and shortest paths (Saha et al., 2021)] to comprehensively characterize the topological information of uncertain brain networks, thus fuse multi-feature to further improve classification validity of the model. At the subgraph selection level, we calculated the discriminative score of frequent subgraphs through the statistical index (i.e., mean) value. However, study has shown that the mean index may not be robust to extreme values (Kong et al., 2013). Therefore, in future research, index such as extreme index (Chen, 2014) can be introduced to satisfy the sensitivity of extreme values between subgraph patterns in uncertain brain networks. At the classification model level, we used traditional machine learning——SVM to classify and diagnose depression. In future research, based on the uncertain brain network model, we can introduce deep learning models such as graph neural network (Zhao et al., 2021) to improve brain psychiatric diseases.

## Conclusion

Studies have shown that certain brain networks inevitably lead to the loss of uncertain information with regards to functional connections. Therefore, uncertain brain networks are proposed to represent uncertain information with regards to functional connections. The frequent subgraph mining (MUSE) method and the discriminative subgraph method (DUG) cannot effectively extract sufficient subgraph features, thus leading to low classification accuracy in the existing uncertain brain network studies. Therefore, in the present study, we used the unFEPG method to mine frequent subgraphs and used the dfsSI method to select discriminative subgraphs from the perspective of probability, in which uncertain information in the uncertain brain network was fully used to improve the ability to identify differences between the MDD and NC groups. The result showed that the unFEPG and dfsSI method obtained a higher classification accuracy. In addition, to further verify the efficacy of the method proposed in this study, we adopted weighting and thresholding methods to unify the subgraph pattern between the uncertain network and the certain network. The classification performance of the uncertain network was superior to that of the certain network within a defined sparsity range. This meant that a satisfactory effect can be obtained from a certain brain network irrespective of whether a higher threshold or an uncertain brain network model was selected. Moreover, if the uncertain brain network model was selected, it is necessary to make full use of the uncertain information held by its functional connections.

## Data Availability Statement

The raw data supporting the conclusions of this article will be made available by the authors, without undue reservation.

## Ethics Statement

The studies involving human participants were reviewed and approved by the Medical Ethics Committee of Shanxi Province (reference number: 2012013). The patients/participants provided their written informed consent to participate in this study.

## Author Contributions

YL was responsible for the study design and writing the manuscript. ZZ, QL, and TL performed the statistical analysis. IJ integrated the experimental data. HG and JC provided the conception and design of the work. All the authors approved the final version of the manuscript.

## Conflict of Interest

The authors declare that the research was conducted in the absence of any commercial or financial relationships that could be construed as a potential conflict of interest.

## Publisher’s Note

All claims expressed in this article are solely those of the authors and do not necessarily represent those of their affiliated organizations, or those of the publisher, the editors and the reviewers. Any product that may be evaluated in this article, or claim that may be made by its manufacturer, is not guaranteed or endorsed by the publisher.

## Footnotes

1
http://www.fil.ion.ucl.ac.uk/spm

2
https://trendscenter.org/software/gift/

3
http://www.cis.hut.fi/projects/ica/icasso

4
http://www.csie.ntu.edu.tw/~cjlin/libsvm/

5
https://bicr-resource.atr.jp/decnefpro/

## References

[B1] Abouelseoud A. Starck T. Remes J. Nikkinen J. Tervonen O. Kiviniemi V. (2010). The effect of model order selection in group PICA. *Hum. Brain Mapp.* 31 1207–1216. 10.1002/hbm.20929 20063361 PMC6871136

[B2] Allen E. A. Erhardt E. B. Damaraju E. Gruner W. Segall J. M. Silva R. F. (2011). A baseline for the multivariate comparison of resting-state networks. *Front. Syst. Neurosci.* 5:2. 10.3389/fnsys.2011.00002 21442040 PMC3051178

[B3] An H. Rajeev O. Huang D. Yang J. Li J. Yu F. (2015). Influence of internal carotid artery stenosis, blood pressure, glycated hemoglobin, and hemoglobin level on fMRI signals of stroke patients. *Neurol. Res.* 37 502–509. 10.1179/1743132815Y.0000000004 25591421

[B4] Beckmann C. F. DeLuca M. Devlin J. T. Smith S. M. (2005). Investigations into resting-state connectivity using independent component analysis. *Philos. Trans. R. Soc. B Biol. Sci.* 360 1001–1013. 10.1098/rstb.2005.1634 16087444 PMC1854918

[B5] Ben-Aharon O. Magnezi R. Leshno M. Goldstein D. A. (2019). Median survival or mean survival: which measure is the most appropriate for patients, physicians, and policymakers? *Oncologist* 24 1469–1478. 10.1634/theoncologist.2019-0175 31320502 PMC6853128

[B6] Calhoun V. D. Kiehl K. A. Pearlson G. D. (2008). Modulation of temporally coherent brain networks estimated using ICA at rest and during cognitive tasks. *Hum. Brain Mapp.* 29 828–838. 10.1002/hbm.20581 18438867 PMC2649823

[B7] Cao B. Kong X. Yu P. S. (2015a). A review of heterogeneous data mining for brain disorder identification. *Brain Inf.* 2 253–264. 10.1007/s40708-015-0021-3 27747561 PMC4883173

[B8] Cao B. Zhan L. Kong X. Yu P. S. Vizueta N. Altshuler L. L. (2015b). “Identification of discriminative subgraph patterns in fMRI brain networks in bipolar affective disorder,” in *Brain Informatics and Health*, eds Guo Y. Friston K. Aldo F. Hill S. Peng H. (Cham: Springer International Publishing), 105–114. 10.1007/978-3-319-23344-4_11

[B9] Chapelle O. Vapnik V. Bousquet O. Mukherjee S. (2002). Choosing multiple parameters for support vector machines. *Mach. Learn.* 46 131–159. 10.1023/A:1012450327387

[B10] Chen E. J. (2014). Range statistics and equivalence tests. *J. Simul.* 8 143–150. 10.1057/jos.2013.23

[B11] Chen Y. Wang C. Zhu X. Tan Y. Zhong Y. (2015). Aberrant connectivity within the default mode network in first-episode, treatment-naïve major depressive disorder. *J. Affect. Disord.* 183 49–56. 10.1016/j.jad.2015.04.052 26001663

[B12] Chen Y. Zhao X. Lin X. Wang Y. Guo D. (2019). Efficient mining of frequent patterns on uncertain graphs. *IEEE Trans. Knowl. Data Eng.* 31:1. 10.1109/TKDE.2018.2830336

[B13] Corbetta M. Shulman G. L. (2002). Control of goal-directed and stimulus-driven attention in the brain. *Nat. Rev. Neurosci.* 3 201–215. 10.1038/nrn755 11994752

[B14] Cui X. Xiang J. Guo H. Yin G. Zhang H. Lan F. (2018). Classification of Alzheimer’s disease, mild cognitive impairment, and normal controls with subnetwork selection and graph kernel principal component analysis based on minimum spanning tree brain functional network. *Front. Comput. Neurosci.* 12:31. 10.3389/fncom.2018.00031 29867424 PMC5954113

[B15] de Ridder M. Klein K. Yang J. Yang P. Lagopoulos J. Hickie I. (2019). An uncertainty visual analytics framework for fMRI functional connectivity. *Neuroinformatics* 17 211–223. 10.1007/s12021-018-9395-8 30099703

[B16] Driver I. D. Whittaker J. R. Bright M. G. Muthukumaraswamy S. D. Murphy K. (2016). Arterial CO_2_ fluctuations modulate neuronal rhythmicity: implications for MEG and fMRI studies of resting-state networks. *J. Neurosci.* 36 8541–8550. 10.1523/JNEUROSCI.4263-15.2016 27535903 PMC4987431

[B17] Du J. Wang L. Jie B. Zhang D. (2016). Network-based classification of ADHD patients using discriminative subnetwork selection and graph kernel PCA. *Comput. Med. Imaging Graph.* 52 82–88. 10.1016/j.compmedimag.2016.04.004 27166430

[B18] Du Y. Fan Y. (2013). Group information guided ICA for fMRI data analysis. *Neuroimage* 69 157–197. 10.1016/j.neuroimage.2012.11.008 23194820

[B19] Erhardt E. B. Rachakonda S. Bedrick E. J. Allen E. A. Adali T. Calhoun V. D. (2011). Comparison of multi-subject ICA methods for analysis of fMRI data. *Hum. Brain Mapp.* 32 2075–2095. 10.1002/hbm.21170 21162045 PMC3117074

[B20] Farahani F. V. Karwowski W. Lighthall N. R. (2020). Application of graph theory for identifying connectivity patterns in human brain networks: a systematic review. *Front. Neurosci.* 13:585. 10.3389/fnins.2019.00585 31249501 PMC6582769

[B21] First M. B. Gibbon M. (1997). *User’s Guide for the Structured Clinical Interview for DSM-IV Axis I Disorders: SCID-1 Clinician Version.* Washington, DC: American Psychiatric Pub.

[B22] Franceschelli M. Giua A. Pisano A. (2017). Finite-time consensus on the median value with robustness properties. *IEEE Trans. Automat. Control* 62 1652–1667. 10.1109/TAC.2016.2590602

[B23] Gao C. Wang J. (2010). “Direct mining of discriminative patterns for classifying uncertain data,” in *Proceedings of the 16th ACM SIGKDD International Conference on Knowledge Discovery and Data Mining* (Washington, DC: Association for Computing Machinery), 861–870. 10.1145/1835804.1835913

[B24] Garrison K. A. Scheinost D. Finn E. S. Shen X. Constable R. T. (2015). The (in)stability of functional brain network measures across thresholds. *Neuroimage* 118 651–661. 10.1016/j.neuroimage.2015.05.046 26021218 PMC4554838

[B25] Graham S. Bellmore A. Nishina A. Juvonen J. (2009). “It Must Be Me”: ethnic diversity and attributions for peer victimization in middle school. *J. Youth Adolesc.* 38 487–499. 10.1007/s10964-008-9386-4 19636723

[B26] Grill-Spector K. Malach R. (2004). The human visual cortex. *Annu. Rev. Neurosci.* 27 649–677. 15217346 10.1146/annurev.neuro.27.070203.144220

[B27] Guo H. Yan P. Cheng C. Li Y. Chen J. Xu Y. (2018). fMRI classification method with multiple feature fusion based on minimum spanning tree analysis. *Psychiatry Res. Neuroimaging* 277 14–27. 10.1016/j.pscychresns.2018.05.001 29793077

[B28] Guo H. Zhang F. Chen J. Xu Y. Xiang J. (2017). Machine learning classification combining multiple features of a hyper-network of fMRI data in Alzheimer’s disease. *Front. Neurosci.* 11:615. 10.3389/fnins.2017.00615 29209156 PMC5702364

[B29] Guy W. (1976). ECDEU Assessment Manual for Psychopharmacology. Rockville: Public Health Service, Alcohol, Drug Abuse, and Mental Health Administration.

[B30] Hagler D. J. Hatton S. Cornejo M. D. Makowski C. Fair D. A. Dick A. S. (2019). Image processing and analysis methods for the adolescent brain cognitive development study. *Neuroimage* 202:116091. 10.1016/j.neuroimage.2019.116091 31415884 PMC6981278

[B31] Hamdi S. M. Aydin B. Boubrahimi S. F. Angryk R. Krishnamurthy L. C. Morris R. (2018). “Biomarker detection from fMRI-based complete functional connectivity networks,” in *Proceedings of the 2018 IEEE First International Conference on Artificial Intelligence and Knowledge Engineering (AIKE)* (Piscataway, NJ: IEEE), 17–24. 10.1109/AIKE.2018.00011

[B32] Huan J. Wang W. Prins J. (2003). “Efficient mining of frequent subgraphs in the presence of isomorphism,” in *Proceedings of the 3rd IEEE International Conference on Data Mining (ICDM 2003)* (Piscataway, NJ: IEEE), 549–552. 10.1109/ICDM.2003.1250974

[B33] Jackson T. S. Read N. (2010a). Theory of minimum spanning trees. I. Mean-field theory and strongly disordered spin-glass model. *Phys. Rev. E Stat. Nonlin. Soft Matter Phys.* 81:021130. 10.1103/PhysRevLett.85.840 20365553

[B34] Jackson T. S. Read N. (2010b). Theory of minimum spanning trees. II. Exact graphical methods and perturbation expansion at the percolation threshold. *Phys. Rev. E Stat. Nonlin. Soft Matter Phys.* 81:021131. 10.1103/PhysRevE.81.021131 20365554

[B35] Jafri M. J. Pearlson G. D. Stevens M. Calhoun V. D. (2008). A method for functional network connectivity among spatially independent resting-state components in schizophrenia. *Neuroimage* 39 1666–1681. 10.1016/j.neuroimage.2007.11.001 18082428 PMC3164840

[B36] Jie B. Zhang D. Wee C.-Y. Dinggang S. (2014). Topological graph kernel on multiple thresholded functional connectivity networks for mild cognitive impairment classification. *Hum. Brain Mapp.* 35 2876–2897. 10.1002/hbm.22353 24038749 PMC4116356

[B37] Jin N. Wang W. (2011). “LTS: discriminative subgraph mining by learning from search history,” in *Proceedings of the 2011 IEEE 27th International Conference on Data Engineering* (Hannover: IEEE), 207–218.

[B38] Ke X. Khan A. Hasan M. A. Rezvansangsari R. (2020). Reliability maximization in uncertain graphs. *IEEE Trans. Knowl. Data Eng.* 1. [Epub ahead of print]. 10.1109/TKDE.2020.2987570

[B39] Khan A. Ye Y. Chen L. (2018b). On uncertain graphs. *Synth. Lect. Data Manage.* 10 1–94. 10.2200/S00862ED1V01Y201807DTM048

[B40] Khan A. Bonchi F. Gullo F. Nufer A. (2018a). “Conditional reliability in uncertain graphs,” in *Proceedings of the IEEE Transactions on Knowledge and Data Engineering* (Piscataway, NJ: IEEE), 2078–2092. 10.1109/TKDE.2018.2816653

[B41] Koechlin E. Ody C. Kouneiher F. (2003). The architecture of cognitive control in the human prefrontal cortex. *Science* 302 1181–1185. 10.1126/science.1088545 14615530

[B42] Koechlin E. Summerfield C. (2007). An information theoretical approach to prefrontal executive function. *Trends Cogn. Sci.* 11 229–235. 10.1016/j.tics.2007.04.005 17475536

[B43] Kong X. Fan W. Yu P. S. (2011). “Dual active feature and sample selection for graph classification,” in *Proceedings of the 17th ACM SIGKDD International Conference on Knowledge Discovery and Data Mining*, San Diego, CA, 654–662. 10.1145/2020408.2020511

[B44] Kong X. Yu P. S. (2014). Brain network analysis: a data mining perspective. *ACM SIGKDD Explor. Newslett.* 15 30–38. 10.1145/2641190.2641196

[B45] Kong X. Yu P. S. Wang X. Ragin A. B. (2013). “Discriminative feature selection for uncertain graph classification,” in *Proceedings of the 2013 SIAM International Conference on Data Mining* (Philadelphia, PA: SIAM), 82–93. 10.1137/1.9781611972832.10 PMC4418485 25949925

[B46] Krienen F. M. Buckner R. L. (2009). Segregated fronto-cerebellar circuits revealed by intrinsic functional connectivity. *Cereb. Cortex* 19 2485–2497. 10.1093/cercor/bhp135 19592571 PMC2742600

[B47] Kudela M. Harezlak J. Lindquist M. A. (2017). Assessing uncertainty in dynamic functional connectivity. *Neuroimage* 149 165–177. 10.1016/j.neuroimage.2017.01.056 28132931 PMC5384341

[B48] Li C. Wang H. de Haan W. Stam C. J. Van Mieghem P. (2011). The correlation of metrics in complex networks with applications in functional brain networks. *J. Stat. Mech. Theory Exp.* 11:11018. 10.1088/1742-5468/2011/11/p11018

[B49] Li F. Xie W. Jiang Y. Fan Z. (2020). “A comparative study of uncertain knowledge representation methods,” in *Proceedings of the 2020 IEEE 4th Information Technology, Networking, Electronic and Automation Control Conference (ITNEC)*, Chongqing, 2038–2042. 10.1109/ITNEC48623.2020.9084983

[B50] Li J. Kong R. Liégeois R. Orban C. Tan Y. Sun N. (2019). Global signal regression strengthens association between resting-state functional connectivity and behavior. *Neuroimage* 196 126–141. 10.1016/j.neuroimage.2019.04.016 30974241 PMC6585462

[B51] Li J. Zou Z. Gao H. (2012). Mining frequent subgraphs over uncertain graph databases under probabilistic semantics. *VLDB J.* 21 753–777. 10.1007/s00778-012-0268-8

[B52] Moreno-Ortega M. Prudic J. Rowny S. Patel G. H. Kangarlu A. Lee S. (2019). Resting state functional connectivity predictors of treatment response to electroconvulsive therapy in depression. *Sci. Rep.* 9:5071. 10.1038/s41598-019-41175-4 30911075 PMC6433903

[B53] Nenert R. Allendorfer J. B. Szaflarski J. P. (2014). A model for visual memory encoding. *PLoS One* 9:e107761. 10.1371/journal.pone.0107761 25272154 PMC4182671

[B54] Nouinou S. Afia A. E. Fkihi S. E. (2018). “Overview on last advances of feature selection,” in *Proceedings of the International Conference on Learning and Optimization Algorithms: Theory and Applications* (Rabat: Association for Computing Machinery), 1–6. 10.1007/978-3-319-67588-6_1

[B55] Papapetrou O. Ioannou E. Skoutas D. (2011). “Efficient discovery of frequent subgraph patterns in uncertain graph databases,” in *Proceedings of the 14th International Conference on Extending Database Technology* (Uppsala: ACM), 355–366.

[B56] Pinto J. Nunes S. Bianciardi M. Dias A. Silveira L. M. Wald L. L. (2017). Improved 7 Tesla resting-state fMRI connectivity measurements by cluster-based modeling of respiratory volume and heart rate effects. *Neuroimage* 153 262–272. 10.1016/j.neuroimage.2017.04.009 28392488 PMC5535271

[B57] Potamias M. Bonchi F. Gionis A. Kollios G. (2010). k-nearest neighbors in uncertain graphs. *Proc. VLDB Endow.* 3 997–1008. 10.14778/1920841.1920967

[B58] Prokopiou P. C. Pattinson K. T. S. Wise R. G. Mitsis G. D. (2018). Modeling of dynamic cerebrovascular reactivity to spontaneous and externally induced CO_2_ fluctuations in the human brain using BOLD-fMRI. *Neuroimage* 186 533–548. 10.1016/j.neuroimage.2018.10.084 30423427

[B59] Riaz A. Asad M. Alonso E. Slabaugh G. (2020). DeepFMRI: end-to-end deep learning for functional connectivity and classification of ADHD using fMRI. *J. Neurosci. Methods* 335:108506. 10.1016/j.jneumeth.2019.108506 32001294

[B60] Richardson M. (2010). Current themes in neuroimaging of epilepsy: brain networks, dynamic phenomena, and clinical relevance. *Clin. Neurophysiol.* 121 1153–1175. 10.1016/j.clinph.2010.01.004 20185365

[B61] Saha A. Brokkelkamp R. Velaj Y. Khan A. Bonchi F. (2021). Shortest paths and centrality in uncertain networks. *Proc. VLDB Endow.* 14 1188–1201. 10.14778/3450980.3450988

[B62] Seifritz E. Esposito F. Hennel F. Mustovic H. Neuhoff J. G. Bilecen D. (2002). Spatiotemporal pattern of neural processing in the human auditory cortex. *Science* 297 1706–1708. 10.1126/science.1074355 12215648

[B63] Sen B. Mueller B. Klimes-Dougan B. Cullen K. Parhi K. K. (2019). “Classification of major depressive disorder from resting-state fMRI,” in *Proceedings of the 2019 41st Annual International Conference of the IEEE Engineering in Medicine & Biology Society (EMBC)* (Piscataway, NJ: IEEE), 3511–3514. 10.1109/EMBC.2019.8856453 31946635

[B64] Shao W. Peng Y. Zu C. Wang M. Zhang D. (2020). Hypergraph based multi-task feature selection for multimodal classification of Alzheimer’s disease. *Comput. Med. Imaging Graph.* 80:101663. 10.1016/j.compmedimag.2019.101663 31923610

[B65] Smith S. M. Fox P. T. Miller K. L. Glahn D. C. Fox P. M. Mackay C. E. (2009). Correspondence of the brain’s functional architecture during activation and rest. *Proc. Natl. Acad. Sci. U.S.A.* 106:13040. 10.1073/pnas.0905267106 19620724 PMC2722273

[B66] Specht K. Reul J. (2006). Functional segregation of the temporal lobes into highly differentiated subsystems for auditory perception: an auditory rapid event-related fMRI-task. *Neuroimage* 20 169–173. 10.1016/j.neuroimage.2003.07.034 14683700

[B67] Sporns O. (2011). The human connectome: a complex network. *Ann. N. Y. Acad. Sci.* 1224 109–125. 10.1111/j.1749-6632.2010.05888.x 21251014

[B68] Sporns O. (2018). Graph theory methods: applications in brain networks. *Dialogues Clin. Neurosci.* 20 111–121. 10.31887/DCNS.2018.20.2/osporns 30250388 PMC6136126

[B69] Stam C. J. Tewarie P. Van Dellen E. Van Straaten E. C. W. Hillebrand A. Van Mieghem P. (2014). The trees and the forest: characterization of complex brain networks with minimum spanning trees. *Int. J. Psychophysiol.* 92 129–138. 10.1016/j.ijpsycho.2014.04.001 24726900

[B70] Steardo L. Carbone E. A. Filippis R. D. Pisanu C. Fazio P. D. (2020). Application of support vector machine on fMRI data as biomarkers in schizophrenia diagnosis: a systematic review. *Front. Psychiatry* 11:588. 10.3389/fpsyt.2020.00588 32670113 PMC7326270

[B71] Steiner A. R. Rousseau-Blass F. Schroeter A. Hartnack S. Bettschart-Wolfensberger R. (2020). Systematic review: anaesthetic protocols and management as confounders in rodent blood oxygen level dependent functional magnetic resonance imaging (BOLD fMRI)–part a: effects of changes in physiological parameters. *Front. Neurosci.* 14:577119. 10.3389/fnins.2020.577119 33192261 PMC7646331

[B72] Tanaka S. C. Yamashita A. Yahata N. Itahashi T. Lisi G. Yamada T. (2021). A multi-site, multi-disorder resting-state magnetic resonance image database. *Sci. Data* 8:227. 10.1038/s41597-021-01004-8 34462444 PMC8405782

[B73] Tewarie P. Van D. E. Hillebrand A. Stam C. J. (2015). The minimum spanning tree: an unbiased method for brain network analysis. *Neuroimage* 104 177–188. 10.1016/j.neuroimage.2014.10.015 25451472

[B74] Tokuda T. Yoshimoto J. Shimizu Y. Okada G. Takamura M. Okamoto Y. (2018). Identification of depression subtypes and relevant brain regions using a data-driven approach. *Sci. Rep.* 8:14082. 10.1038/s41598-018-32521-z 30237567 PMC6148252

[B75] Tong Y. Hocke L. M. Frederick B. B. (2019). Low frequency systemic hemodynamic “Noise” in resting state BOLD fMRI: characteristics, causes, implications, mitigation strategies, and applications. *Front. Neurosci.* 13:787. 10.3389/fnins.2019.00787 31474815 PMC6702789

[B76] Vakamudi K. Posse S. Jung R. Cushnyr B. Chohan M. O. (2019). Real-time presurgical resting-state fMRI in patients with brain tumors: quality control and comparison with task-fMRI and intraoperative mapping. *Hum. Brain Mapp.* 41 797–814. 10.1002/hbm.24840 31692177 PMC7268088

[B77] Van Dellen E. Sommer I. E. Bohlken M. M. Tewarie P. Draaisma L. Zalesky A. (2018). Minimum spanning tree analysis of the human connectome. *Hum. Brain Mapp.* 39 2455–2471. 10.1002/hbm.24014 29468769 PMC5969238

[B78] Vincent J. L. Kahn I. Snyder A. Z. Raichle M. E. Buckner R. L. (2008). Evidence for a frontoparietal control system revealed by intrinsic functional connectivity. *J. Neurophysiol.* 100 3328–3342. 10.1152/jn.90355.2008 18799601 PMC2604839

[B79] Wang Q. Poh J. S. Wen D. J. Broekman B. F. P. Chong Y.-S. Yap F. (2019). Functional and structural networks of lateral and medial orbitofrontal cortex as potential neural pathways for depression in childhood. *Depress. Anxiety* 36 365–374. 10.1002/da.22874 30597677

[B80] Wig G. S. (2017). Segregated systems of human brain networks. *Trends Cogn. Sci.* 21 981–996. 10.1016/j.tics.2017.09.006 29100737

[B81] Williams J. B. (1988). A structured interview guide for the Hamilton Depression Rating Scale. *Arch. Gen. Psychiatry* 45 742–747. 10.1001/archpsyc.1988.01800320058007 3395203

[B82] Yamashita A. Sakai Y. Yamada T. Yahata N. Kunimatsu A. Okada N. (2020). Generalizable brain network markers of major depressive disorder across multiple imaging sites. *PLoS Biol.* 18:e3000966. 10.1371/journal.pbio.3000966 33284797 PMC7721148

[B83] Yan X. (2002). “gSpan: graph-based substructure pattern mining,” in *Proceedings of the 2002 IEEE International Conference on Data Mining* (Piscataway, NJ: IEEE), 721–724. 10.1109/ICDM.2002.1184038

[B84] Yan X. Cheng H. Han J. Yu P. S. (2008). “Mining significant graph patterns by leap search,” in *Proceedings of the 2008 ACM SIGMOD International Conference on Management of Data (SIGMOD)*, Vancouver, BC, 433–444. 10.1145/1376616.1376662

[B85] Yuan Y. Wang G. Chen L. Ning B. (2016). Efficient pattern matching on big uncertain graphs. *Inf. Sci.* 339 369–394. 10.1016/j.ins.2015.12.034

[B86] Zhang D. Huang J. Jie B. Du J. Tu L. Liu M. (2018). Ordinal pattern: a new descriptor for brain connectivity networks. *IEEE Trans. Med. Imaging* 37 1711–1722. 10.1109/TMI.2018.2798500 29969421

[B87] Zhang Y. Lin H. Yang Z. Wang J. Liu Y. (2017). An uncertain model-based approach for identifying dynamic protein complexes in uncertain protein-protein interaction networks. *BMC Genomics* 18(Suppl. 7):743. 10.1186/s12864-017-4131-6 29513194 PMC5657050

[B88] Zhang Y. Wu W. Toll R. T. Naparstek S. Etkin A. (2021). Identification of psychiatric disorder subtypes from FC patterns in rsEEG. *Nat. Biomed. Eng.* 5 309–323. 10.1038/s41551-020-00614-8 33077939 PMC8053667

[B89] Zhao F. Chen Z. Rekik I. Lee S. W. Shen D. (2020). Diagnosis of autism spectrum disorder using central-moment features from low- and high-order dynamic resting-state functional connectivity networks. *Front. Neurosci.* 14:258. 10.3389/fnins.2020.00258 32410930 PMC7198826

[B90] Zhao K. Duka B. Xie H. Oathes D. J. Calhoun V. Zhang Y. (2021). A dynamic graph convolutional neural network framework reveals new insights into connectome dysfunctions in ADHD. *Neuroimage* 246:118774. 10.1016/j.neuroimage.2021.118774 34861391 PMC10569447

[B91] Zhou H.-X. Chen X. Shen Y.-Q. Li L. Chen N.-X. Zhu Z.-C. (2020). Rumination and the default mode network: meta-analysis of brain imaging studies and implications for depression. *Neuroimage* 206:116287. 10.1016/j.neuroimage.2019.116287 31655111

[B92] Zhou Z. Chen X. Zhang Y. Qiao L. Yu R. Pan G. (2020). Brain network construction and classification toolbox (BrainNetClass). *Hum. Brain Mapp.* 41 2808–2826. 10.1002/hbm.24979 32163221 PMC7294070

[B93] Zou Z. Li J. Gao H. Zhang S. (2009). “Frequent subgraph pattern mining on uncertain graph data,” in *Proceeding of the 18th ACM Conference on Information and Knowledge Management, CIKM‘09*, Hong Kong, 583–592. 10.1145/1645953.1646028

[B94] Zou Z. Li J. Gao H. Zhang S. (2010). Mining frequent subgraph patterns from uncertain graph data: mining large uncertain and probabilistic databases. *IEEE Trans. Knowl. Data Eng.* 22 1203–1218. 10.1109/TKDE.2010.80

