# Supplementary material for: Depression Classification Using Frequent Subgraph Mining Based on Pattern Growth of Frequent Edge in Functional Magnetic Resonance Imaging Uncertain Network
Source: Front Neurosci. 2022 Apr 29;16:889105. doi: 10.3389/fnins.2022.889105 (PMC9106560; doi:10.3389/fnins.2022.889105)
Supplement: Supplementary file 1 [file Image_1.pdf]

Supplemental Figure S1. Differences in the discriminative capabilities of subgraphs between the uncertain network and the certain network.

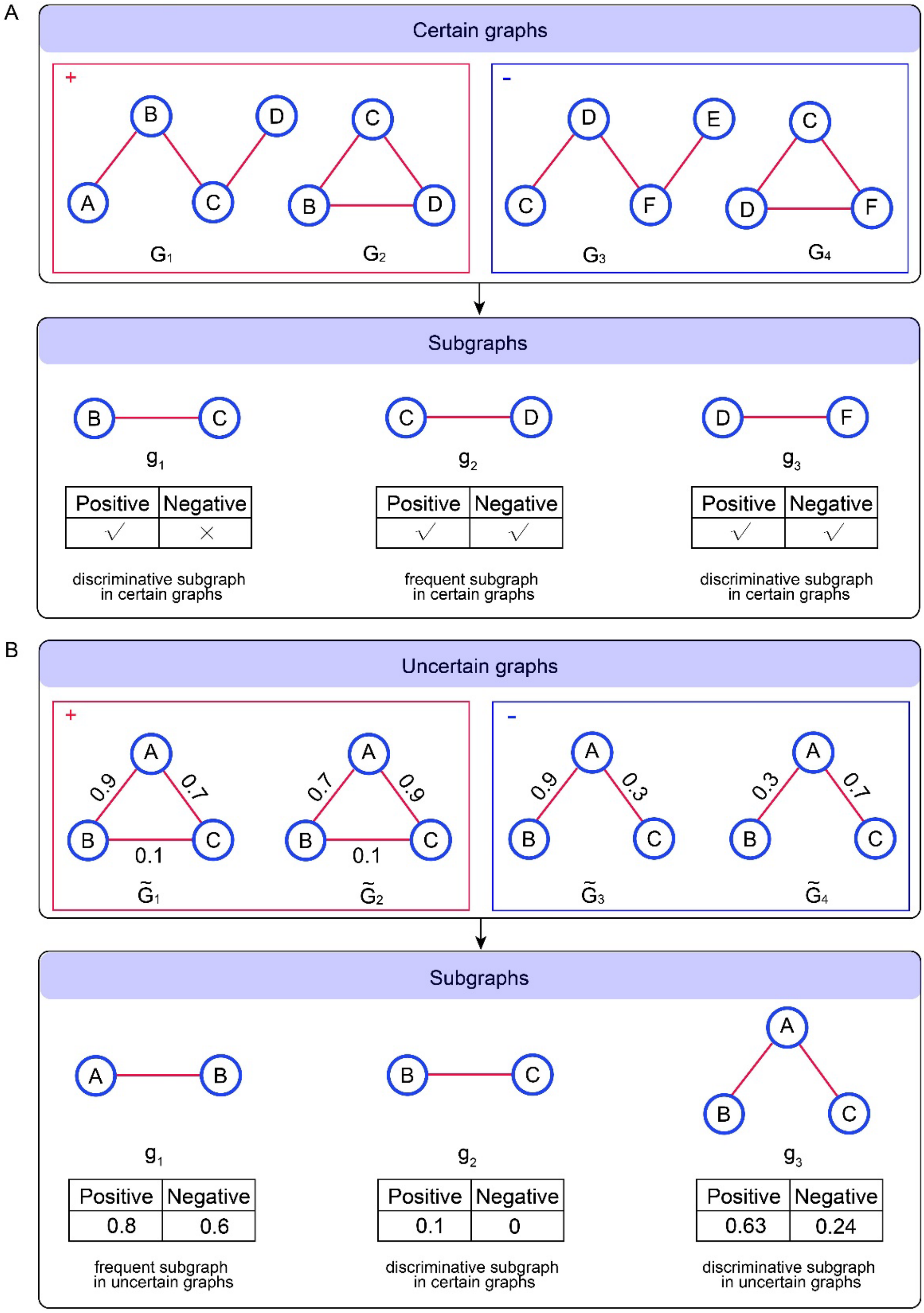

Fig. 1. **Differences in the discriminative capabilities of subgraphs between the uncertain network and the certain network.** Fig. 1A shows a certain graph dataset including four certain graphs $G_1,\ldots,G_4$ with their class labels, + or -. '+' representing positive samples; '-' represents negative samples. A, B,..., F refer to nodes in the graph. Subgraph g_1 only occurs in positive samples and does not occur in negative samples.

Subgraph g_2 occurs in both positive samples and negative samples. Subgraph g_3 only occurs in negative samples and does not occur in positive samples. Subgraph g_1 and g_3 than g_2 is more suitable as a discriminative subgraph in certain graphs to perform classification. Fig. 3B shows an uncertain graph dataset including four uncertain graphs $\tilde{G}_1, ..., \tilde{G}_4$ with their class labels, + or -. '+' representing positive samples; '-' represents negative samples. It is assumed that the *minsup* in frequent subgraph mining is 0.2. On this basis, subgraph g_1 is a frequent subgraph among the uncertain graphs but may not be related to the class labels of the graphs (the expected support degree for positive samples = 0.8; for negative samples, this is 0.6). Subgraph g_2 is a discriminative subgraph if we neglect the edge uncertainties. However, when such uncertainties are considered, we find that g_2 is rarely observed in the uncertain graph data set. Furthermore, this cannot be considered as a frequent subgraph in uncertain graphs (positive samples: 0.1; negative samples: 0). Subgraph g_3 is a frequent subgraph among uncertain graphs and is related to the class labels of the graphs (positive samples: 0.63; negative samples: 0.24). Thus, this not only represents a frequent graph, it also represents a discriminative subgraph for uncertain graphs.
