# Supplementary material for: Depression Classification Using Frequent Subgraph Mining Based on Pattern Growth of Frequent Edge in Functional Magnetic Resonance Imaging Uncertain Network
Source: Front Neurosci. 2022 Apr 29;16:889105. doi: 10.3389/fnins.2022.889105 (PMC9106560; doi:10.3389/fnins.2022.889105)
Supplement: Supplementary file 2 [file Image_2.pdf]

Supplemental Figure S2. Spatial maps of the 22 components identified as intrinsic connection network (ICNs).

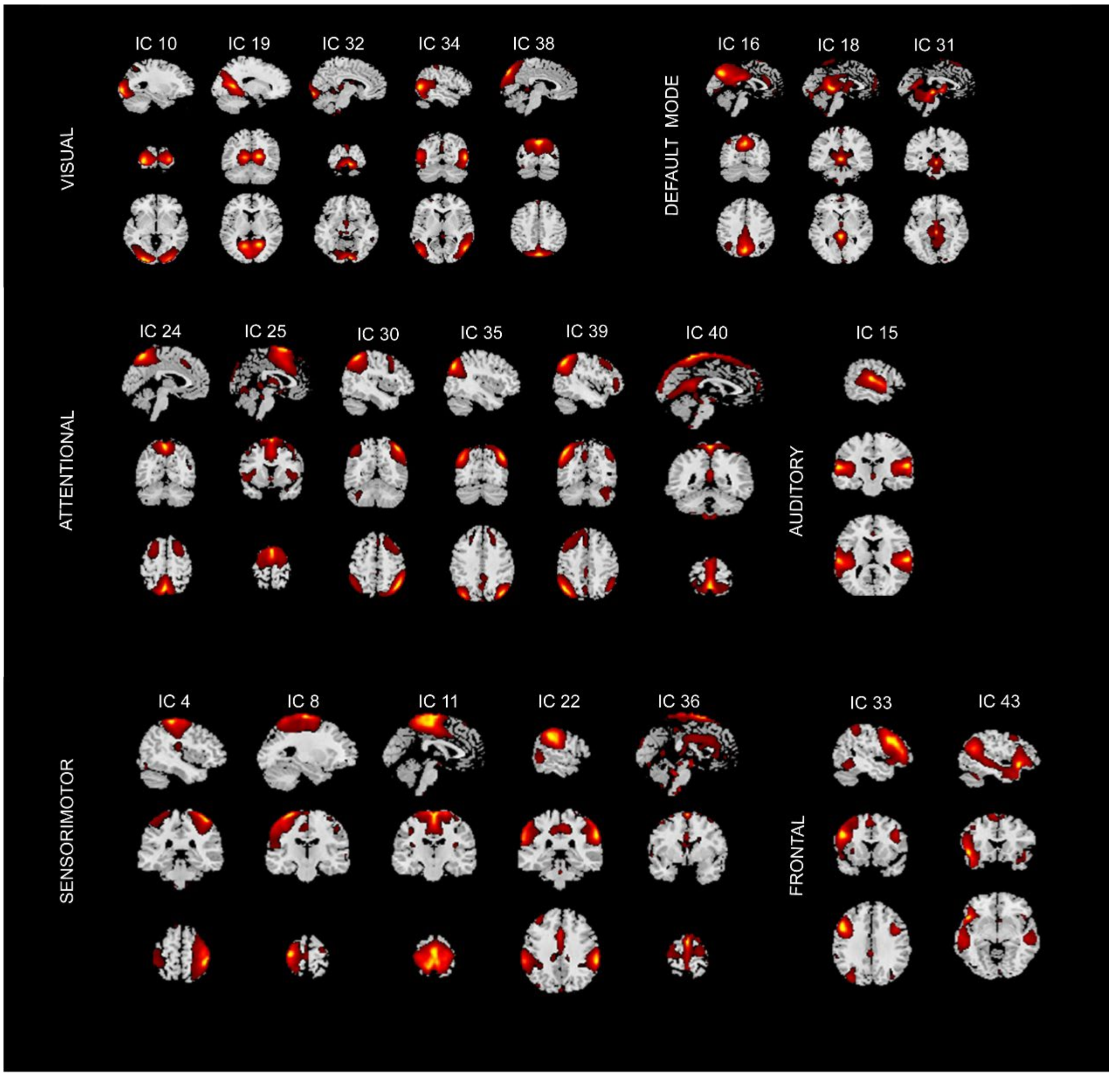

Fig. S2. **Illustrates Spatial maps of the 22 components identified as ICNs.** Spatial maps are plotted for the three most informative slices as *t*-statistics. ICNs are divided into groups based on their anatomical and functional properties within each of the following networks: AUD, SM, VIS, DMN, ATTN, and FRONT. AUD, auditory network; SM, sensorimotor network; VIS, visual network; DMN, default mode network; ATTN, attentional network; FRONT, frontal network.
