# Supplementary material for: Depression Classification Using Frequent Subgraph Mining Based on Pattern Growth of Frequent Edge in Functional Magnetic Resonance Imaging Uncertain Network
Source: Front Neurosci. 2022 Apr 29;16:889105. doi: 10.3389/fnins.2022.889105 (PMC9106560; doi:10.3389/fnins.2022.889105)
Supplement: Supplementary file 4 [file Table_2.docx]

**Supplemental Table S2. The information of frequent subgraphs**

| NC | | MDD | |
| --- | --- | --- | --- |
| edges | **The number of subgraphs** | **edges** | **The number of edges** |
| 1 | 147 | 1 | 117 |
| 2 | 123 | 2 | 72 |
| 3 | 15 | 3 | 3 |
| 4 | 4 |  | - |

NC is normal control group and MDD is major depression disorder group. Edges indicate how many edges are contained in a frequent subgraph. If the frequent subgraph contains 1 edge, the subgraph belongs to the 1-subgraph pattern.
