# Supplementary material for: Depression Classification Using Frequent Subgraph Mining Based on Pattern Growth of Frequent Edge in Functional Magnetic Resonance Imaging Uncertain Network
Source: Front Neurosci. 2022 Apr 29;16:889105. doi: 10.3389/fnins.2022.889105 (PMC9106560; doi:10.3389/fnins.2022.889105)
Supplement: Supplementary file 5 [file Data_Sheet_1.docx]

**Supplemental Text S1. The power analysis of subjects**

Power analysis can [examine](https://fanyi.sogou.com/?keyword=examine&fr=websearch_submit&from=en&to=zh-CHS) the sample size needed for a given effect size when degree of confidence is fixed, which is related to four quantities: sample size, significance level, power, and effect size. In current study, GPower toolbox [1] was introduced to carry out power analysis. The t-test was selected as test family. Means-difference between two independent means (two groups) was selected as statistical test. A priori (sample size N is computed as a function of power level 1 *- b*, significance level *a*, and the to-be-detected population effect size) was selected as the type of statistical power analysis. The significance level and power were specified as 5%.and 80%. The effect size was calculated by discriminative subgraph feature data between MDD group and the NC group. Finally, enter the ratio of MDD and NC in our study. By computing, based on number of datasets in current study, actual power reached 81.79%. However, in general, when the efficacy value is 80%, the experiment is considered reasonable. Therefore, it was indicated the size of the sample is appropriate in our study.

**Reference**

1. Faul, F., E. Erdfelder, A.G. Lang, et al., G*Power 3: A flexible statistical power analysis program for the social, behavioral, and biomedical sciences[J]*.* Behav Res Methods, 2007. 39(2): p. 175-191.
