# Supplementary material for: Depression Classification Using Frequent Subgraph Mining Based on Pattern Growth of Frequent Edge in Functional Magnetic Resonance Imaging Uncertain Network
Source: Front Neurosci. 2022 Apr 29;16:889105. doi: 10.3389/fnins.2022.889105 (PMC9106560; doi:10.3389/fnins.2022.889105)
Supplement: Supplementary file 6 [file Data_Sheet_2.docx]

**Supplemental Text S2. Image acquisition.**

All the subjects underwent resting state functional MRI scan using 3T MR equipment (Siemens Trio 3-Tesla scanner，Siemens, Erlangen, Germany). During the scan, subjects were instructed to relax with their eyes closed but not to fall asleep. Subjects were fitted with soft ear plugs and positioned carefully in the coil with comfortable support.

Each scan consisted of 248 contiguous EPI functional volumes (33 axial slices, repetition time (TR) = 2000 ms, echo time (TE) = 30 ms, thickness/skip = 4/0 mm, field of view (FOV) = 192×192 mm, matrix = 64×64 mm, flip angle = 90°) and the first ten volumes of time series were discarded for magnetization stabilization.
