# Supplementary material for: Depression Classification Using Frequent Subgraph Mining Based on Pattern Growth of Frequent Edge in Functional Magnetic Resonance Imaging Uncertain Network
Source: Front Neurosci. 2022 Apr 29;16:889105. doi: 10.3389/fnins.2022.889105 (PMC9106560; doi:10.3389/fnins.2022.889105)
Supplement: Supplementary file 7 [file Data_Sheet_3.docx]

**Supplemental Text S3. The rationality used for selecting 54 ICs**

For ICA, extensive feature is the uncertainty of the number of independent components (ICs) [1]. A review was listed including 47 recent studies on ICA in fMRI datasets. Some of these studies were reviewed in [1] and [2]. Additional researches published in the past four years (2016–2020) were included (See Supplemental Table S1). Through summary, it is found that there is no gold standard for determining the numbers of ICs. In different studies, the numbers of ICs used vary widely, up to 150 ICs and at least 8 ICs. Among them, there are 16 articles with ICs greater than 50.

Moreover, through summary, it is found that GIFT toolbox and MELODIC (multivariate exploratory linear decomposition) toolbox were commonly used to determine the number of independent components based on fMRI data in existing studies, of which there were 27(57.45%) studies that selected GIFT toolbox to calculate the number of independent components. The minimum description length criterion (MDL) was adopted in the GIFT. Considering the spatial correlation, the number of best decomposed components in all subjects was automatically estimated. In this process, mainly based on the input of pre-processed image data, directly through a specific algorithm to pre-determine the number of the best components, rather than artificial settings. Further, It was considered appropriate that when optimal number of ICs is approximate one-fourth to one-fifth of the number of the time points [3]. In our experiment, after removing the first 10 time points, there were 238 remaining time points, so the appropriate range of independent components was 47-59, 54 components (number of ICs identified in our study) being located in it. Because the independent components extracted using group independent component analysis included not only brain network components of interest in this paper, but also other unrelated components or components with more noise. Because of this, the study was further screened and confirmed by prior template matching method and manual inspection method. Finally, 32 unrelated components were removed and 22 components were retained to participate in the post-study.

**Reference**

[1] Wang, Y. and T.Q. Li, Dimensionality of ICA in resting-state fMRI investigated by feature optimized classification of independent components with SVM[J]*.* Frontiers in Human Neuroscience. 9.

[2] Dennis, E.L. and P.M. Thompson, Functional Brain Connectivity Using fMRI in Aging and Alzheimer’s Disease[J]*.* Neuropsychology Review. 24(1): p. 49-62.

[3] Greicius, M.D., G. Srivastava, A.L. Reiss, et al., Default-mode network activity distinguishes Alzheimer's disease from healthy aging: evidence from functional MRI[J]*.* Proceedings of the National Academy of Sciences, 2004. 101(13): p. 4637-4642.
