# Supplementary material for: Depression Classification Using Frequent Subgraph Mining Based on Pattern Growth of Frequent Edge in Functional Magnetic Resonance Imaging Uncertain Network
Source: Front Neurosci. 2022 Apr 29;16:889105. doi: 10.3389/fnins.2022.889105 (PMC9106560; doi:10.3389/fnins.2022.889105)
Supplement: Supplementary file 8 [file Data_Sheet_4.docx]

**Supplemental Text S4. An example about unFEPG algorithm.**

We took a subgraph in Figure 2 as an example to describe the flow of the unFEPG algorithm and the frequent subgraphs obtained. This subgraph was shown in Figure 1 below. Specific process was shown in Figure 2 below.


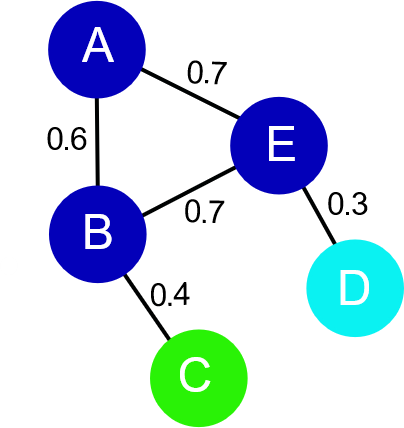


**Fig. 1. A graph.** The node includes {A, B, C, D, E}. The value of edge represents the probability value of each edge..


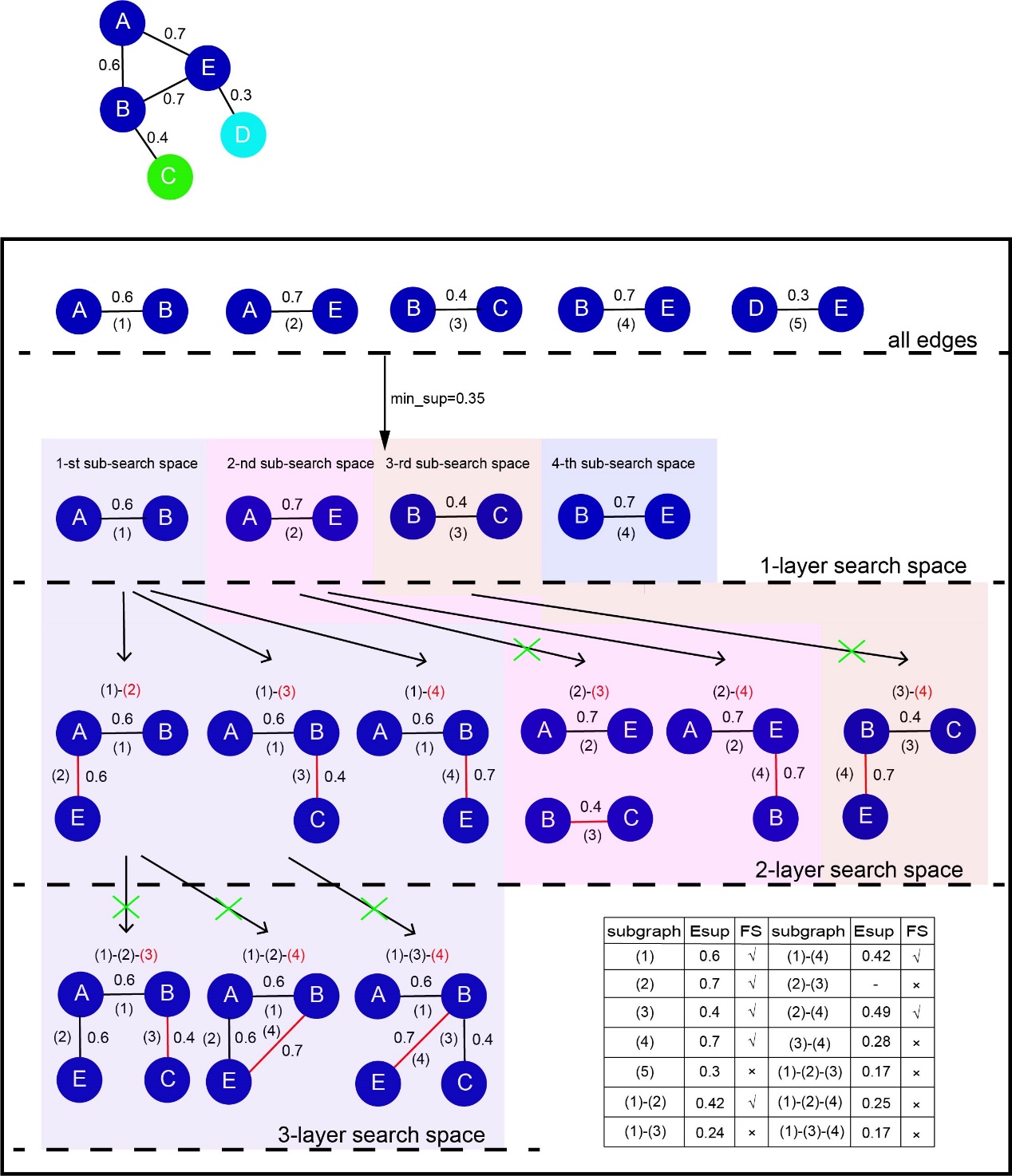


**Fig. 2. Specific process about unFEPG algorithm.** The *min_sup* represents minimum support degree. FS represents frequent subgraph. Esup represents the expected support degree in the subgraph. The (*i*) (*i*=1,2,...,5) represents edge label. Red edges represent frequent edges added on the basis of previous subgraph pattern set. The ‘×’ represents this subgraph iss not a frequent subgraph and is pruned. Different colored backgrounds indicate different sub-search spaces. A subgraph of one edge is a 1-layer search space. A subgraph of two edges is a 2-layer search space. A subgraph of three edges is a 3-layer search space.
