# Supplementary material for: Depression Classification Using Frequent Subgraph Mining Based on Pattern Growth of Frequent Edge in Functional Magnetic Resonance Imaging Uncertain Network
Source: Front Neurosci. 2022 Apr 29;16:889105. doi: 10.3389/fnins.2022.889105 (PMC9106560; doi:10.3389/fnins.2022.889105)
Supplement: Supplementary file 9 [file Data_Sheet_5.docx]

**Supplemental Text S5. Frequent subgraph mining and feature selection methods for the certain network.**

Definition 1(Certain graph) Certain graph$G=\left(V,E\right)$ is an undirected and deterministic graph, which is represented as a set of nodes$V=\left\{v_{1},v_{2},...,v_{n_{v}}\right\}$ and a set of deterministic edges $E\subseteq V\times V$.

Definition 2 (frequency) $G=\left\{G_{1},G_{2},......G_{n}\right\}$is a certain graph and the number of subgraphs is n, where the frequency of subgraphs is defined as the following formula:

$$f_{q}(\mathrm{g}_{s}|\mathrm{G})=\frac{|\mathrm{g}_{s} \mathrm{is} \mathrm{subgraph} \mathrm{of} \mathrm{G}_{i} \mathrm{and} \mathrm{G}_{i}\in G|}{|\mathrm{G}|}$$

**Method for mining frequent subgraphs (gSpan)**

The detailed algorithm is shown in Table 1.

| Input: |
| --- |
| certain network set $G$ and support degree $s$. |
| 1. A lexicographic order among graphs is constructed by using the gSpan algorithm *s* and employing a unique minimum depth-first search (DFS) code as the canonical label. |
| 2. Judgment of whether the k-subgraph candidate mode is a frequent subgraph. From an initial one-edge frequent subgraph mode, gradually increase the number of edges and determine if this mode is a frequent subgraph. |
| 3. If the k+1-subgraph is non-frequent, delete it from the candidate subgraph. |
| Output: |
| the set of frequent subgraphs. |

**Frequent-scoring feature selection.**

The discriminant subgraph features can be used for classification. However, the subgraph obtained by the gSpan algorithm is not discriminative. In particular, some frequent subgraphs have weak discriminative ability, and the resulting classification performance is not good. Therefore, it is necessary to define the discriminant and use it as an index to select the most discriminative subgraphs as features. This method is called frequent-scoring feature selection.

Given the positive sample set $G_{p}=\{\mathrm{g}_{p1},\mathrm{g}_{p2,...,}\mathrm{g}_{pm}\}$ and the negative sample set $G_{n}=\{\mathrm{g}_{n1},\mathrm{g}_{n2},...,\mathrm{g}_{nm}\}$, the discrimination score is defined as

$$S(\mathrm{g}_{s})=|\mathrm{f}_{q}(\mathrm{g}_{s}|\mathrm{G}_{p})-\mathrm{f}_{q}(\mathrm{g}_{s}|\mathrm{G}_{n})|$$

the difference between its positive frequency and negative frequency. A larger score reflects a larger difference between the two groups.

In this paper, this method involved choosing the same number of frequent subgraphs from both the NC and the MDD groups, calculating and sorting the discrimination scores of the frequent subgraphs, and then selecting top *k* subgraphs. As a result, a total of *2k* subgraphs were selected.
