# Supplementary material for: Depression Classification Using Frequent Subgraph Mining Based on Pattern Growth of Frequent Edge in Functional Magnetic Resonance Imaging Uncertain Network
Source: Front Neurosci. 2022 Apr 29;16:889105. doi: 10.3389/fnins.2022.889105 (PMC9106560; doi:10.3389/fnins.2022.889105)
Supplement: Supplementary file 10 [file Data_Sheet_6.docx]

**Supplemental Text S6. Discussion on discriminant components from the perspective of brain regions**

The best classification performance was obtained when 30 frequent subgraph patterns were selected as discriminative subgraph patterns. Therefore, we analyzed the most discriminative abnormal components obtained by 30 discriminative subgraphs. By counting the number of occurrences of each independent component in all subgraphs, the top three components were considered to be the most discriminative components (IC16, IC32 and IC34) in the diagnosis of depression. Of these, IC16 located in the default mode network, which included mainly precuneus and angular gyrus. In existing researches, these regions are associated with the pathogenesis of depression. Melinda et al. [1] found Depression——induced suicidality was positively associated with resting state functional connectivity between precuneus and middle frontal gyri etc. Findings of hyperconnectivity stemming from the precuneus may reflect maladaptive self-reflection and mentalization. Wei et al. [2] performed the first fully voxel-level resting state functional-connectivity neuroimaging analysis of depression of the precuneus and found Functional connectivity was also increased in depression between the precuneus and angular areas. The remaining two discriminative components, IC32 and IC34, were contained in the visual network, but IC 34 mainly included middle temporal gyrus and middle occipital gyrus. Similarly, these regions have also been proved to be relevant to the pathological mechanisms of depression in existing studies. For example, Cao et al. [3] proposed a feature selection method to classify the depression and normal. The result showed that middle temporal gyrus and middle occipital gyrus play important roles in discrimination process. IC32 mainly included inferior parietal, but supramarginal and angular gyri, middle frontal gyrus, median cingulate and paracingulate gyri. Similarly, based on rs-fMRI data, Yu et al.[4] applied Hilbert-Huang transform method to the abnormal brain areas of patients with depression. The result indicated that supramarginal and angular gyri, middle frontal gyrus, median cingulate and paracingulate gyri are potential biomarker that reflects the pathophysiological abnormalities of depression. In addition, Liu et al. [5] showed CACNA1C Gene rs11832738 polymorphism influences depression severity by modulating spontaneous activity in the right middle frontal gyrus in patients with major depressive disorder. Therefore, according to brain regions, it could also be concluded that the markers of depression in current study were the same as the existing research.

1. M.W. Schreiner, B. Klimes-Dougan, and K.R. Cullen, Neural Correlates of Suicidality in Adolescents with Major Depression: Resting-State Functional Connectivity of the Precuneus and Posterior Cingulate Cortex*.* *Suicide and Life-Threatening Behavior*, **49**(2019), 899-913. doi: https://doi.org/10.1111/sltb.12471.

2. E.T. Rolls, W. Cheng, J. Du, D. Wei, J. Qiu, D. Dai, et al., Functional connectivity of the right inferior frontal gyrus and orbitofrontal cortex in depression*.* *Social Cognitive and Affective Neuroscience*, **15**(2020), 75-86. doi: 10.1093/scan/nsaa014.

3. M.D. Longlong Cao, S. Guo, Z.X.M. PhD, H. Yong, M.D. Haihong Liu, T.E. Mwansisya, et al., Aberrant functional connectivity for diagnosis of major depressive disorder: A discriminant analysis*.* *Psychiatry & Clinical Neurosciences*, **68**(2014), 110–119.

4. H. Yu, F. Li, T. Wu, R. Li, L. Yao, C. Wang, et al., Functional brain abnormalities in major depressive disorder using the Hilbert-Huang transform*.* *Brain Imaging & Behavior*, **12**(2018), 1556-1568. doi: 10.1007/s11682-017-9816-6.

5. X. Liu, Z. Hou, Y. Yin, C. Xie, H. Zhang, H. Zhang, et al., CACNA1C Gene rs11832738 Polymorphism Influences Depression Severity by Modulating Spontaneous Activity in the Right Middle Frontal Gyrus in Patients With Major Depressive Disorder*.* *Frontiers in Psychiatry*, **11**(2020), doi: 10.3389/fpsyt.2020.00073.
