# Supplementary material for: Depression Classification Using Frequent Subgraph Mining Based on Pattern Growth of Frequent Edge in Functional Magnetic Resonance Imaging Uncertain Network
Source: Front Neurosci. 2022 Apr 29;16:889105. doi: 10.3389/fnins.2022.889105 (PMC9106560; doi:10.3389/fnins.2022.889105)
Supplement: Supplementary file 11 [file Data_Sheet_7.docx]

**Supplemental Text S7. Verification of generalization performance for classifiers by the proposed method**

We verified the generalization performance of the proposed method from two aspects. On the one hand, we divided our datasets into a training set and a validation set (they are the same site), where the validation set did not participate in the construction of the classification model at all and did not participate in the process of subgraph feature extraction and selection, but was used directly to validate classification model. On the other hand, we introduced independent validation datasets from other sites and used them to evaluate the generalization performance of classification models.

The generalization performance of the classification results of our dataset is shown in Table 1 below.

Table 1 Verification of generalization performance under different methods in our dataset

| Method | Research | Accuracy (%) | Sensitivity (%) | Specificity (%) |
| --- | --- | --- | --- | --- |
| Frequent subgraph mining of uncertain graphs | PV-FC | 75.17 | 79.90 | 71.02 |
|  | unFEPG method | 83.72 | 97.73 | 62.71 |
|  | nFEPG and dfsSI method | 89.56 | 92.28 | 87.00 |
|  | DUG method | 78.06 | 83.27 | 77.24 |
| Frequent subgraph mining of certain graphs based on thresholding method | Sparsity=0.05 | 68.28 | 77.64 | 44.74 |
|  | Sparsity=0.10 | 78.69 | 91.69 | 59.18 |
|  | Sparsity=0.15 | 80.02 | 83.54 | 78.00 |
|  | Sparsity=0.20 | 86.14 | 88.73 | 84.25 |
|  | Sparsity=0.25 | 84.04 | 86.53 | 80.31 |
|  | Sparsity=0.30 | 86.81 | 89.15 | 83.29 |
|  | Sparsity=0.35 | 91.02 | 92.54 | 90.00 |
|  | Sparsity=0.40 | 90.75 | 94.49 | 85.15 |

The PV-FC method represents probability values representing functional connections. The unFEPG method represents frequent subgraph pattern mining algorithm based on pattern growth of frequent edge. The unFEPG and dfsSI method represents combining frequent subgraph pattern mining algorithm based on pattern growth of frequent edge and discriminative feature selection method based on statistical index. The DUG method represents the traditional discriminative feature selection for uncertain graph classification algorithm. MDD, major depressive disorder.

The generalization performance of the classification results of open dataset is shown in Table 2 below. The results show that the classifier constructed in this paper has reached more than 70% on all independent data sets, and the accuracy in the HUH dataset was the highest, reaching 75%, which is about 17% lower than that of the classification performance obtained in our dataset (92.90%). The underlying reason might be that there are differences in the data acquired by different imaging centers, such as measurements bias generated by differences in fMRI parameters and MR scanners, and sampling bias due to recruiting subjects, which will result in a lower generalization accuracy than that obtained from the same imaging center [1]. Moreover, we did not further reduce site difference using harmonization method. In addition, in the independent validation dataset, the nodes of each brain network were defined by a priori templates (*i.e*. the digital atlas of the Brainvisa Sulci Atlas), while we adopted a data-driven approach———independent component analysis to divided nodes in our dataset. Therefore, the definition of different nodes might also cause differences in generalization performance. The main reason why we did not perform independent component analysis in independent validation dataset is that independent component analysis defines nodes according to the data itself [2]. There were different independent components in different datasets, so even if the independent components were defined, it could not be guaranteed that the estimated components were completely consistent with our dataset. Besides, after estimating the independent components, selecting effective independent components requires a lot of time to read a large number of relevant references. Therefore, considering the time constraints, we adopted a priori template method to divide the independent validation dataset. But, in general, the generalization performance of the classifier constructed in this paper was acceptable.

Moreover, compared with the existing research, the results were higher than the results in the existing research. This indicated that the classifier constructed with the proposed method in this paper was effective.

Table 2 The classification performance based on independent validation dataset

| Site |  | Accuracy (%) | Sensitivity (%) | Specificity (%) | BAC (%) |
| --- | --- | --- | --- | --- | --- |
| HUH | Yamashita et al. [3] | 59 | 65 | 53 | - |
|  | unFEPG and dfsSI method | 75 | 67 | 81 | 74 |
| HRC | Yamashita et al. [3] | 68 | 81 | 63 | - |
|  | unFEPG and dfsSI method | 73 | 68 | 78 | 73 |
| HKH | Yamashita et al. [3] | 57 | 63 | 50 | - |
|  | unFEPG and dfsSI method | 72 | 72 | 72 | 72 |
| COI | Yamashita et al. [3] | - | - | - | - |
|  | unFEPG and dfsSI method | 70 | 62 | 79 | 72 |
| All | Yamashita et al. [3] | 66 | 72 | 61 | - |
|  | unFEPG and dfsSI method | 73 | 69 | 78 | 74 |

HUH: Hiroshima University Hospital; HRC: Hiroshima Rehabilitation Center; HKH: Hiroshima Kajikawa Hospital COI: Center of Innovation in Hiroshima University; All: all datasets. BAC: balanced accuracy.

**References**

1. B. Rashid and V. Calhoun, Towards a brain-based predictome of mental illness*.* *Human Brain Mapping*, **41**(2020), 3468-3535. doi: https://doi.org/10.1002/hbm.25013.

2. V. Schöpf, C. Windischberger, C.H. Kasess, R. Lanzenberger, and E. Moser, Group ICA of resting-state data: a comparison*.* *Magnetic Resonance Materials in Physics, Biology and Medicine*, **23**(2010), 317-325. doi: 10.1007/s10334-010-0212-0.

3. A. Yamashita, Y. Sakai, T. Yamada, N. Yahata, A. Kunimatsu, N. Okada, et al., Generalizable brain network markers of major depressive disorder across multiple imaging sites*.* *PLOS Biology*, **18**(2020), e3000966. doi: 10.1371/journal.pbio.3000966.
